# Validation and Clinical Applications of a Comprehensive Next Generation Sequencing System for Molecular Characterization of Solid Cancer Tissues

**DOI:** 10.3389/fmolb.2019.00082

**Published:** 2019-09-25

**Authors:** Mehdi Dehghani, Kevin P. Rosenblatt, Lei Li, Mrudula Rakhade, Robert J. Amato

**Affiliations:** ^1^Division of Oncology, Department of Internal Medicine, The University of Texas Health Science Center at Houston McGovern Medical School, Houston, TX, United States; ^2^NX Prenatal, Inc., Bellaire, TX, United States; ^3^Consultative Genomics, PLLC, Bellaire, TX, United States; ^4^Principle Health Systems, Houston, TX, United States

**Keywords:** solid tumor, molecular profiling, next-generation sequencing, analytical validation, Ion AmpliSeq

## Abstract

Identification of somatic molecular alterations in primary and metastatic solid tumor specimens can provide critical information regarding tumor biology and its heterogeneity, and enables the detection of molecular markers for clinical personalized treatment assignment. However, the optimal methods and target genes for clinical use are still being in development. Toward this end, we validated a targeted amplification-based NGS panel (Oncomine comprehensive assay v1) on a personal genome machine sequencer for molecular profiling of solid tumors. This panel covers 143 genes, and requires low amounts of DNA (20 ng) and RNA (10 ng). We used 27 FFPE tissue specimens, 10 cell lines, and 24 commercial reference materials to evaluate the performance characteristics of this assay. We also evaluated the performance of the assay on 26 OCT-embedded fresh frozen specimens (OEFF). The assay was found to be highly specific (>99%) and sensitive (>99%), with low false-positive and false-negative rates for single-nucleotide variants, indels, copy number alterations, and gene fusions. Our results indicate that this is a reliable method to determine molecular alterations in both fixed and fresh frozen solid tumor samples, including core needle biopsies.

## Introduction

Cancers are products of micro-evolutionary processes; each cancer patient harbors a unique pattern of molecular alterations in oncogenes and tumor suppressors that together cause aberrant cell signaling that leads to cancer development and progression (Gerlinger et al., 2014; Martincorena and Campbell, 2015). Recent advances in genotyping technologies such as high-throughput deep sequencing have led to the identification of frequent somatic molecular alterations in solid tumor cancers (Lawrence et al., 2013, 2014; Vogelstein et al., 2013; Hovelson et al., 2015). These advances have led to a paradigm shift in cancer companion diagnostics work-ups, from single gene–based tests to multiplexed next-generation sequencing (NGS)–based assays (Cronin and Ross, 2011; Coonrod et al., 2012; Koboldt et al., 2013; Aftimos et al., 2014).

Profiling of the molecular alterations in primary and metastatic tumor tissues can be used to guide therapy selection and to suggest new uses for existing FDA-approved drugs, potential new combinations, and salvage regimens for those who have already failed standard-of-care treatment (Devarakonda et al., 2013; Garraway, 2013; Sleijfer et al., 2013). NGS also enables the establishment of a tumor-agnostic program to study tumor heterogeneity among and within patients—for example, the dissimilar molecular alterations that are often found at different metastatic sites within the same patient.

Molecular tumor profiling is the key to better understanding of the pathogenesis and prognosis of solid tumors. Finding novel molecular alterations that affect certain signaling pathway(s) can lead to the development of new targeted treatment options. Tumor molecular profiling can be explored at different levels; DNA and/or RNA for detecting different types of molecular alteration such as single nucleotide variants (SNVs), insertion/deletions (indels), copy number alterations (CNAs), and fusion transcripts (Hovelson et al., 2015; Luthra et al., 2017).

Amplification-based and hybrid capture-based chemistries for targeted resequencing of tens to hundreds of cancer-related genes, as well as whole exome, genome, and transcriptome sequencing, can be used to study molecular profiles of cancer patients (Beadling et al., 2013; Frampton et al., 2013; Hadd et al., 2013; Singh et al., 2013; Van Allen et al., 2014; Zhang et al., 2014; Hovelson et al., 2015). Depending on the chemistry used for sequencing and the size of target regions of the sequencing assay, the quantity of amplifiable DNA required may differ (Gagan and Van Allen, 2015). The performance of these assays for detecting molecular alterations with low allelic fractions may also vary.

Most existing tissue banks contain formalin-fixed, paraffin-embedded (FFPE) tissues. Molecular profiling can be performed on these tissues, however, formalin fixation can negatively affect DNA amplification because DNA fragments maybe too short. The quality of DNA is inversely proportional to the age of the specimen. Therefore, using fresh tissues, when available, is a preferable option. These might also be an important consideration when comprehensive profiling of different types of molecular alterations (such as SNV, indel, CNA, and fusion transcripts) is performed and only a small amount of biopsy tissue is available in FFPE cores (Hadd et al., 2013).

Comprehensive molecular profiling of cancer biopsies, particularly in a high-throughput laboratory, requires expertise to establish a robust pipeline for quality control, data analysis, and interpretation of actionable variants (Hadd et al., 2013; Rehm et al., 2013). The implementation of these advanced technologies in a clinical laboratory, particularly for the detection of low-allelic-fraction somatic mutations and treatment stratification, faces challenges at the pre-analytical, analytical, and post-analytical levels. A limited amount of tissue obtained from needle biopsies or fine needle aspirates requires a robust nucleic extraction protocol and library construction chemistry compatible with a low input of nucleic acids. In addition, depending on the size of an NGS assay, thousands to millions of bases of the genome are assessed; therefore, robust and optimized analytical settings are required to detect low-allelic-fraction mutations. NGS assay validation in a clinical laboratory not only characterizes the performance and suitability of the molecular assay, but also provides valuable information about the approximate amount of tumor content and tissue size required for successful and reliable sequencing of specimens. Analytical validation can also help with defining and verifying the reportable range of the assay. In regard to NGS, the reportable range is defined as a subset of the targeted regions from which different types of variant can be reliably detected (Santani et al., 2017). Depending on the target enrichment chemistry and the type of sequencer, a fraction of the targeted regions may not be sequenced reliably and thus should be defined and excluded from the reportable range. Regions prone to high systematic errors can be defined based on technical limitations of an NGS platform and validation data. For instance, variants in homopolymer regions, genes with high homology to pseudogenes or within repetitive regions are some of the sources for systematic errors (Bragg et al., 2013; Bayrak et al., 2014; Damiati et al., 2016).

**Figure 1:**
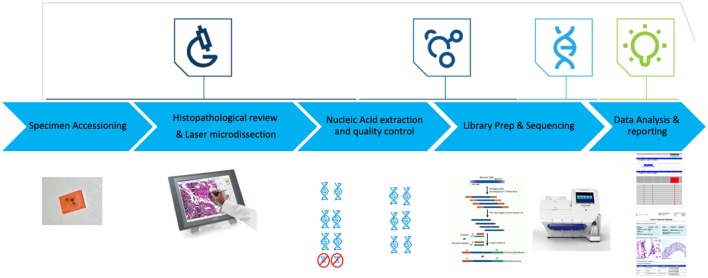
OCAv1 test workflow. Hematoxylin and Eosin-stained slides were reviewed to ensure each specimen contained adequate tumor content. Laser microdissection was used to enrich tumor content to ≥50%. DNA and RNA were co-purified, then their quality and quantity were assessed. Libraries created from the corresponding DNA and RNA specimens were sequenced on the personal genome machine. Sequence data were analyzed and reviewed using the Ion Reporter software.

Here, we report the analytical validation of the Oncomine Comprehensive Cancer assay version 1 (OCAv1), a multiplexed and scalable amplification-based NGS assay, and a pre-analytical to post-analytical pipeline to detect known activating mutations in the oncogenes and deleterious mutations in tumor suppressor genes covered by this panel and to report potential treatment strategies (Figure 1). The OCAv1 NGS panel is agnostic to solid tumor type and was designed based on genomic data from ~700,000 tumor specimens and frequent driver copy number and fusion alterations reported previously (Hovelson et al., 2015; Luthra et al., 2017). Thus, it has a potential to provide insight into treatment strategies based on actionable variants.

We also conducted an initial registry study with the objective of developing a tumor-specific molecular classification database using tissue from planned routine biopsies or surgeries for building a database to correlate clinical and histopathological assessments with the molecular profile for each solid tumor system.

## Methods

### Reference Materials Used for Validation

Commercially available reference materials were used to evaluate the performance of the OCAv1 DNA panel. The AcroMetrix® Oncology Hotspot Ladder (AOHL; ThermoFisher) was used to assess the analytical sensitivity (lowest limit of detection, LLOD) of the assay for detecting somatic mutations.

The NIST RM8398 human DNA for whole-genome variant assessment (https://www-s.nist.gov/srmors/reports/8398.pdf?CFID=19090737&CFTOKEN=f85957fdf11df514-E85BE885-C6D3-8DC4-A3229D410A13529C) contains human genomic DNA extracted from a large growth of the human lymphoblastoid cell line GM12878 and is intended to provide a whole human genome sample and accompanying reference values to assess performance of variant calling from genome sequencing. Reference values are provided for SNVs, indels, and reference genotypes for ~77% of the genome. RM8398 was used to evaluate the specificity of the NGS DNA assay for SNVs and indels.

AcroMetrix® MultiMix FFPE controls (ThermoFisher, CA), the quantitative multiplex reference standard (HD200, Horizon Discovery, Cambridge, UK), the EGFR quantitative multiplex FFPE reference standard (HD300, Horizon Discovery, Cambridge, UK), and FFPE samples with known molecular alterations were used to assess the analytical sensitivity of the assay (Tables S1A–C).

ATCC (Manassas, VA) cancer cell lines with matched normal cell lines, cell lines from the Coriell Institute for Medical Research (Camden, NJ), a structural multiplex reference standard (HD753, Horizon Discovery, Cambridge, UK), and an ERBB2 amplification reference standard (HD-C511, Discovery, Cambridge, UK) were used to evaluate the performance of the CNV assay (Tables S1A–C). To mimic FFPE tissues, pellets from cell line cultures were fixed in 10% neutral buffered formalin and embedded in paraffin following a standard protocol.

Three FISH reference standards (HD231, HD640, and HD615), the RNA multiplex ALK-ROS-RET reference standard (HD-C188, Horizon Diagnostics, UK), containing 50% EML-ALK, 22% CCDC6-RET, 63% SLC34A2-ROS, an additional of the three fusions (10% EML4-ALK, 5% SLC34A2-ROS1, and 10% CCDC6-RET), the Vcap cell line which harbors the TMPRSS2-ERG translocation, and clinical FFPE samples with confirmed fusion variants were used to evaluate the performance of the OCAv1 RNA panel (Tables S1A–C).

### Formalin-Fixed Paraffin and OCT-Embedded Tissue Specimens

A total of 27 FFPE samples were sequenced in this study. Fourteen of these samples were previously processed clinical samples for routine molecular testing that demonstrated positive results; therefore, they were included in the validation study. Sanger sequencing was used for detecting mutations in seven genes, including *PTEN, APC, CTNNB1, MYD88, TP53, KRAS*, and *EGFR*. The competitive Allele-Specific Taqman® PCR (castPCR) assay was used for detecting the *BRAF* p.V600E mutation. The Signature KRAS mutation assay (Asuragen, Austin, TX) was used for detecting the *KRAS* p.Gly12Cys or p.Gly12Val mutation. The FISH assay for detecting EML-ALK fusions was performed by the Baylor Miraca Genetics Laboratories (Houston, TX). The *EGFRvIII* variant was confirmed using the Taqman® gene expression assay (see the qRT-PCR and FISH Confirmation section, below). Alteration concordance was evaluated based on the positive variants detected in these 14 samples.

Twenty-six OCT-embedded, fresh frozen (OEFF) specimens from informed consented patients enrolled in the IRB-approved clinical registry at The University of Texas Health Science Center at Houston–Oncology Division were also tested using the OCAv1 test. The protocol (B-14-105) was reviewed and approved by the committee for protection of human subjected at UThealth.

### Pathologic Review

Five-micron sections of hematoxylin and eosin–stained slides were reviewed on-site by certified pathologists to confirm the diagnosis and to ensure each sample had at least 10% tumor cellularity and sufficient tumor content. Laser microdissection (Ion LMD II, Seoul, Korea) was used to enrich tumor content to ≥50%.

### Nucleic Acid Isolation

The Qiagen AllPrep FFPE Kit (Qiagen, Valencia, CA) was used to isolate DNA and RNA per the manufacturer's instructions. DNA and RNA quantity and quality were assessed using a Qubit 2.0 (ThermoFisher, CA) and a Bioanalyzer 2100 (Agilent, CA), respectively.

### Library Preparation for DNA and RNA Panels

OCAv1 includes two pools of primers for the DNA panel and one pool of primers for the RNA panel. These primer pools were used for the preparation of amplicon libraries from DNA and RNA simultaneously. This panel was designed to amplify 2,737 amplicons (2,530 from DNA and 207 from RNA) covering 143 cancer-related genes. The libraries were prepared with the Ion AmpliSeq™ Oncomine Research Panel primer pools using the Ion AmpliSeq™ Library Kit 2.0 (ThermoFisher, CA) following the manufacturer's instructions. Briefly, 20 ng of the genomic DNA isolates were used in two target amplification reactions, which were then combined. For the RNA panel, the libraries were prepared using 10 ng of total RNA and reverse-transcribed, followed by the target amplification reaction. The prepared libraries for the DNA and RNA panels were partially digested and phosphorylated using the FuPa™ reagent, ligated to different barcode adapters, then purified. The purified libraries were quantified using the Ion Library TaqMan™ Quantitation Kit. A bacterial DNA standard provided by the kit was used as a standard for the quantification of the libraries.

### Sequencing

Barcoded DNA and RNA libraries of each individual sample were diluted to 100 pM and combined at a 4:1 ratio for each sample. The template of the pooled libraries of one sample was generated using the Ion PGM™ template OT2 200 Kit on an Ion OneTouch2 system according to the manufacturer's instructions. Template-positive Ion Sphere Particle were enriched, and the sequencing was performed using an Ion 318 chip with the Ion PGM™ Sequencing 200 Kit v2 on the Ion Torrent PGM sequencer according to the manufacturer's instructions.

### Sanger Sequencing

Putative actionable SNVs and indels were confirmed by Sanger sequencing. PCR amplification of the target regions and cycle sequencing reactions were performed using the BigDye Direct Cycle Sequencing Kit protocol recommended by the manufacturer (ThermoFisher). The sequencing products were purified using the BigDye XTerminator Purification Kit (ThermoFisher). Capillary electrophoresis was performed on an Applied Biosystems® 3500 or 3730XL Genetic Analyzer. Sequence data was analyzed using the Mutation Surveyor DNA Variant Analysis Software (v4.0.9) (Softgenetics, State College, PA).

### QRT-PCR and Fish Confirmation

Gene rearrangements were confirmed by TaqMan® gene expression assays designed specifically for each fusion event (see Table S2 for primer and probe sequences). Initially, 10–100 ng total RNA was reverse transcribed into cDNA templates using the SuperScript® VILO™ MasterMix (ThermoFisher). A qRT-PCR reaction included 900 nM each of the two unlabeled primers, 250 nM dye-labeled TaqMan® MGB probe, 1 × TaqMan® gene expression master mix, and 2–100 ng cDNA templates. GAPDH was used as the internal expression control.

### Data Analysis

Data analyses for variant calling (SNVs/ multi-nucleotide variants [MNVs], indels, and fusion transcripts) were performed in Ion Reporter software v4.4 with manufacturer-recommended settings. Data analyses for identifying copy number variations in fresh frozen samples were performed in Ion Reporter software v5.0 with a default baseline and manufacturer recommended settings. For identifying copy number variations in FFPE samples, a custom baseline was generated using run data of six normal male FFPE samples from Biochain (T2234090-D02, Newark, CA) and 73 FFPE cancer tissues according to the manufacturer's instructions, and data were analyzed in Ion Reporter software v5.0 using this baseline and the manufacturer-recommended settings. Grasso et al. (2015) have developed, tested, and validated the algorithm for detecting somatic CNAs in NGS data generated from amplicon-based libraries through comparisons to hybrid-capture library-based sequencing, FISH, and aCGH and that NGS data pooled from multiple normal samples can be substituted for a matched normal tissue without affecting the detection of clinically relevant CNAs. Therefore, a matched normal sample was not required for each tumor sample for data analysis. The CNA detection algorithm has also been functionally validated for the OCAv1 by the manufacturer (ThermoFisher, CA).

## Results

### Analytical Validation

The OCAv1 assay was validated in a single workflow (Figure 1) to evaluate the nucleic acid extraction method, sequencing platform, test, and bioinformatics pipeline. In this validation, the performance characteristics of the assay were assessed.

### OCAv1 Assay Performance Quality Metrics

To ensure reliability of the sequencing data, quality control metrics recommended by vendor, such as DNA and RNA library concentration, total reads of DNA and RNA panels, percentage of uniformity of coverage, percentage of amplicons with >100× coverage (for DNA panel), and MAPD score for each sequencing run were used to reject, cautiously accept, or accept each sequencing run. The recommended thresholds (Table 1) were verified empirically using the data from familiarization study, and then verified by analytical assay validation study.

**Table 1:** OCAv1 assay quality control parameters used and verified in this study.

**Quality control parameter**	**Cutoff**	**<Cutoff action**
DNA library concentration (pmole/l)	20	Fail
RNA library concentration (pmole/l)	20	Fail
Total reads for DNA library (million counts)	3	Flag
Total reads for RNA library (thousand counts)	80	Flag
% U of coverage (DNA)	80	U < 80: Fail, 80 < U < 90: Flag
% amplicon with >100× coverage	90	Fail
MAPD score	0.9	Fail

*U, uniformity*.

The guidelines for validation of NGS-based oncology panels by Jennings et al. recommends the minimum of 250× amplicon coverage to be used for clinical NGS testing for detection of somatic variants. However, this guideline also suggests that the coverage threshold be systematically assessed and validated along with the other parameter settings (Jennings et al., 2017). In order to ensure that, this depth of coverage cutoff is sufficient for detecting SNVs and indels with 10% allelic frequencies, the sequencing data from two runs of hotspot ladder three reference samples (with 361 SNVs, 11 insertions, and 18 deletions with allelic frequencies ~10%) with mapped reads of 3,879,093 and 4,437,680, respectively (Tables S3A,B), were combined and then, down-sampled to two new ^*^.bam files with target mean coverages of 3,000×, 1,500×, 1,000×, 500×, 350×, and 250× each. The 12 ^*^.bam files generated (duplicate for each coverage) were uploaded to IR4.4 and analyzed. The minimal coverages obtained at 250× average coverage for the hotspot SNVs, MNVs, and indels tested are listed in Table S4. At an average depth of coverage of 250×, 99.4% hotspot SNVs and 100% multi-nucleotide polymorphisms were detected. The results also showed that we were able to detect the *EGFR* c.2235_2249del15 variant (15bp deletion, COSM6223) with an observed allele frequency of 11.86% and the *ERBB2* c.2322_2323ins12 alteration (12 bp insertion, COSM682) with an observed allele frequency of 8.43% with 253× and 332× amplicon coverage, respectively. Hovelson et al. (2015) also showed that that the OCAv1 assay is able to detect indels with <10 bases, at allele frequency of ~20% in the custom version of AOHC tested in their study (Hovelson et al., 2015). Based on the down-sampling analysis, it seems that the 250× minimum coverage recommended in the recently published guideline by Jennings et al. (2017) is acceptable for detecting SNVs and Indels with allele fraction of 10 and 20%, respectively. However, to increase the sensitivity of the assay, we implemented a laser microdissection system to enrich the tumor content of clinical samples to at least 50% tumor cellularity.

### Lowest Limit of Detection of OCAv1 for the Detection of SNVs, Indels, Known Fusion Transcripts, and High-Level CNAs

Defining the LLODs for low-allelic-fraction alterations in heterogeneous cancer specimens such as tumor biopsies, where the testing samples contain mixed contents of tumor and normal cells, would provide information to define acceptance criteria for required tumor content or tumor cellularity for the molecular assay. To determine the LLOD of variants or the lowest detectable percentage mutation for different types of alterations, the reference materials given in Tables S1A–C were used. One DNA and one RNA sample were paired to run on a 318 chip to obtain an average depth of >1,500 reads (Tables S3A,B). The paired samples used for studying the LLOD of the assay were run in duplicate.

**Figure 2:**
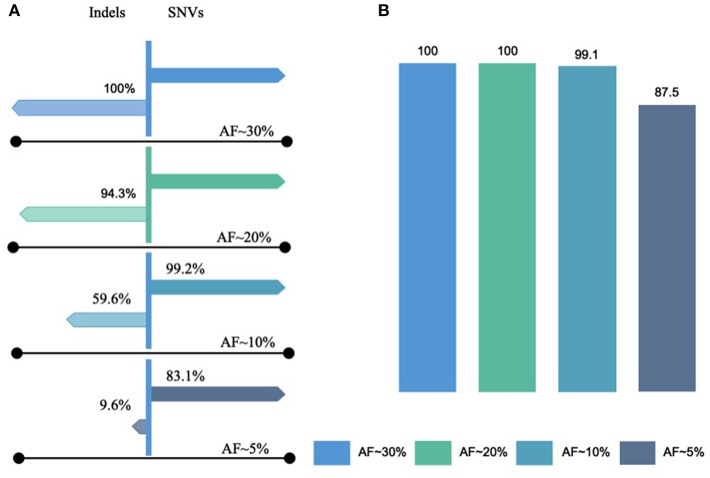
Lowest limits of detection for SNVs and Indels. The lowest limit of detection of OCAv1 for identifying SNVs and indels, with expected allele frequencies of ~2.5–50%, were evaluated using the AOHL member 1 to member 6 ladders and were sequenced. **(A)** Indicates the detection rates of the assay for SNVs and Indels with expected allele frequencies of ~5–30% (hotspot and non-hotspot variants); and, **(B)** shows the detection rate of the assay for variants listed in the hotspot bed file.

To determine the LLOD of SNVs and indels, the AOHL member 1 to member 6, with expected allele frequency of ~2.5–50%, were sequenced with a minimum mapped reads of 3,878,493, a minimum mean depth of coverage of 1,569, a minimum coverage uniformity of 95%, and an average reads per amplicon of 1,496 (Tables S3A,B). The OCAv1 covers 390 engineered variants present in AOHL that are within the reportable range of the DNA assay, including 361 SNVs, 11 insertions, and 18 deletions. As outlined in the Table S5, the assay was able to detect all SNVs with an observed allele frequency of >13.4%, all indels with an observed allele frequency of >17.6%, and all known hotspot variants (with an observed allele frequency of >10.7% for SNVs and 10.3% for indels) that were included in the hotspot analysis bed file, provided by the vendor. As shown in the Figure 2, the detection rate of the assay at ~20% expected allele frequency, based on the sequencing results of hotspot ladder four samples, was overall 99.4%: 100% for SNVs, 94.3% for indels, and 100% for known hotspot mutations. In a previous study, Hovelson et al. (2015) used a custom version of the AcorMetrix Oncology Hotspot Control containing 365 SNVs/MNVs and 33 indels each at an expected allele frequency of 20% to assess the performance of the DNA assay of the OCAv1. The detection rate of their assay at 20% expected allele frequency was reported to be 99.7% for SNVs/MNVs and 75.8% for indels (Hovelson et al., 2015). It should be noted that the reported detection rate in our study only applies to the reportable range of the OCAv1, which explains the higher detection rate reported in this report compared to Hovelson et al. group. The detection rate of our assay at ~10% expected allele frequency was overall 96.4%: 99.2% for SNVs, 59.6% for indels, although it was 99.1% for known hotspot mutations (Figure 2). The detection rate of the assay at 5% expected allele frequency was 78.1% overall: 83.1% for SNVs and 9.6% for indels; however, it was 87.5% for known hotspot mutations (Detailed data can be find in the Table S5). The slight difference in the detection rate at low allele frequency between SNVs and hotspot variants is largely due to the fact that in the Ion Reporter 4.4 OCAv1 analysis workflow, the minimal allele frequency setting (cutoff) for all hotspot variants, is 3%; however, the cutoffs for novel SNVs and indels are 4 and 7%, respectively.

To define the LLOD for CNAs, DNA was extracted from tumor cell lines (ATCC, VA) harboring known CNAs and diluted to tumor purities of 80, 50, 40, 30, and 20% using DNAs from their matching normal cell lines. In addition, libraries were prepared using DNAs extracted from Coriell cell lines and CNA reference materials from Horizon Discovery. These samples cover 28 known CNAs ranging from 0 to 16 copies. As summarized in Table 2, all amplifications with copy numbers ≥8, except for *AKT1*, were detected in samples with 50% expected tumor purity. All of the high-level amplifications (CN ≥ 8) present in HCC1143/HCC1143BL and HCC2218/HCC2218BL tumor-normal pairs at various tumor purity showed decreased CN with the increase in tumor dilution, which was similar to results of a previous study using AmpliSeq® technology on Ion Torrent PGM sequencing platform (Grasso et al., 2015). It is worth noting that in HCC1143 non-fixed cells, the known *AKT1* amplification with an expected CN of 11 was called correctly with an observed CN of 11.34; however, in HCC1143 FFPE cells, this variant was called by our data analysis pipeline but with an observed CN of 6.42. Therefore, we believe that the low sensitivity of detecting *AKT1* amplification in HCC1143/HCC1143BL FFPE cells at various tumor purity was due to DNA degradation produced during formalin fixation process. Based on our results, the detection rate at 50% estimated tumor cellularity for detection of homozygous deletions (CN 0) and amplifications with CN ≥8 were 100 and 87.5%, respectively. Interestingly, the detection rate of the assay for detecting high level CNAs (>8) did not change when the tumor cell fraction percentages changed from 30 to 50%. However, the detection rate at 80% tumor fraction for both the >8 copy CNAs and homozygous deletion were 100% (Detailed data can be found in the Table S6 **“LOD FFPE” and “LOD summary”**).

**Table 2:** The LOD of OCAv1 assay in regards to the lowest amount of tumor cellularity required for the detection of high level CNAs (>8 copies).

**Gene**	**Expected copy number**	**Observed copy number in fresh tumor cell line**	**Observed copy number in FFPE tumor cell line pooled at various tumor cell percentages**					
		**100%**	**100%**	**80%**	**50%**	**40%**	**30%**	**20%**
*CCND1*	12	24.61	13.8 ± 0.44	12.58 ± 0.11	10.1 ± 2.50	8.08 ± 0.02	7.82 ± 0.14	NA
*MYC*	8	7.16	7.27 ± 0.25	6.33 ± 0.13	4.23 ± 0.37	4.53 ± 0.11	4.96 ± 0.08	NA
*AKT1*	11	11.34	6.42 ± 0.19	5.49 ± 0.04	2.57 ± 0.06	2.05 ± 0.09	1.43 ± 0.06	NA
*MDM2*	9	11.27	7.9 ± 0.01	7.18 ± 0.03	6.6 ± 1.04	4.89 ± 0.11	4.34 ± 0.13	NA
*ERBB2*	9	18.77	17.11	16.36	13.48	12.98	13.63	13.1

A validation study done by Frampton et al. (2013) showed 99% analytical sensitivity for high-level amplification (CN ≥8), and homozygous deletion at 30% tumor purity is achieved. Compared with our study, the hybrid capture method was used for target enrichment and library prep, following by sequencing on an Illumine platform. In addition, a different bioinformatics pipeline and algorithm for CNA analysis (allele-specific copy number analysis of tumors, ASCAT) was used (Van Loo et al., 2010; Frampton et al., 2013). However, more importantly, the difference in specimen types used for the validation (fresh pools of cell lines or DNAs vs. FFPE samples) might have contributed greatly to the difference in performance.

To determine the LLOD of the assay for detecting rearrangement/fusions, total RNAs extracted from Horizon Discovery FISH reference slides (HD231) harboring 50% *EML4/ALK* inv(2) (p21;p23) variant 1 were diluted to 20, 10, 5, 2.5, and 1% with total RNA extracted from the GM12878 cell line. This fusion variant was detected in all tested RNA samples to as low as 1% expected allele frequency (Table S7). The LLOD of the assay for detecting CCDC6(1)-RET(12), *SLC34A2*(4)-*ROS1*(32), and *TMPRSS2*(1)-*ERG*(4) fusion transcript were also verified using the same approach on mRNA specimens spiked with mRNA from the HD640 (*CCDC6*(1)-*RET*(12)), HD615 (*SLC34A2*(4)-*ROS1*(32)), Vcap (TMPRSS2(1)-ERG(4)) cell lines. Therefore, the LOD of the assay for detecting fusion events was 1% expected allele frequency in RNA samples (cutoff of the number of fusion reads was set at 20 for known fusions).

Overall, based on the results of this study, the sensitivity of this molecular assay for detecting SNVs with ~10% allele frequency and indels with ~20% allele frequency was determined to be 99.2 and 94.3%, respectively. For known hotspot mutations with an allele frequency of ~10%, the sensitivity of the assay for both hotspot SNVs and indels was >99%. The LLOD of the assay for known variants was also verified with the results of AcroMetrix hotspot control, HorizonDx quantitative multiplex and HorizonDX EGFR multiplex reference samples, where all SNVs (AF >5%) and indels (>10%) in reportable range of the assay were detected. We also showed that this assay can detect fusion transcripts with variant fraction down to 1%.

Luthra et al. evaluated the LLOD of this assay on Ion Proton platform by sequencing DNA from two FFPE tumor samples sequentially diluted (from 50 to 6.25% dilutions) into DNA from FFPE normal brain tissue. One tumor sample was positive for 9 SNVs at various allelic frequencies, and the other had CNAs in five genes. In addition, DNA from FFPE cell lines preparation from CCL-221 and HTB-30 cells, diluted sequentially (50 to 3.125%) into DNA from FFPE HTB-177 cells, were used to complement the LLOD study (Luthra et al., 2017). Based on their study, the LLOD of the assay for detecting SNVs was reported to be ≥5%. However, the LLOD of the assay for indels was not reported in that article. The LLOD of the assay for detecting CNAs was reported to be ≥3, although the reportable cutoff was set to be ≥7.

### Analytical Sensitivity

To define the overall analytical sensitivity of the OCAv1 assay for the detection of SNVs and indels, AcroMetrix Hotspot frequency Ladders 5 and 6 (Table S5), AcroMetrix Hotspot Control, Horizon Discovery Quantitative Multiplex DNA reference standard, Horizon Discovery EGFR Quantitative Multiplex DNA reference standard, AcroMetrix FFPE MultiMix reference standards A-H, 5 non-tumor FFPE samples (Biochain®, CA) that were negative for gain-of-function alterations in *EGFR, BRAF, KRAS*, and *NRAS* genes (confirmed by Oncofocus panel™, On MassArray system; Agena, CA), and 14 FFPE clinical reference samples (Table S8) enriched to tumor cellularity of ≥50% by LMD were evaluated based on the true variants called. Variants detected were compared with those obtained with orthogonal methods.

The commercial version of AOHC (ThermoFisher, CA) used in our study contained 521 engineered variants (all confirmed by Sanger) with expected allele frequencies ranging from 5 to 35%. Among these variants, 361 SNVs and 13 indels were covered by the OCAv1 and above our LLOD. OCAv1 assay was able to detect 100% of these 374 variants. The custom version of AOHC used in a previous OCAv1 validation study (Hovelson et al., 2015) contains 398 variants (365 applicable SNVs/MNVs and 33 indels), each at an expected allele frequency of 20%. Among those variants, 99.7% OCAv1 targeted SNV/MNVs and 75.8% OCAv1 targeted indels have been detected (Hovelson et al., 2015).

Among the 16 known mutations covered by the Quantitative Multiplex Reference Standard (Horizon Discovery, UK), 10 were detected with our default call setting (factory recommended analysis setting in IR4.4) used in this validation study. The same results were obtained by Hovelson et al. (2015) using the same reference material (Hovelson et al., 2015). Among the remaining six variants, two (*BRCA2* p.A1689fs, and *NF1* p.L626fs) resided in areas with excessive errors, which are not included in our reportable range, and four variants had allele frequencies lower than our LLOD. From 19 FFPE patient samples (harboring 7 indels and 16 SNVs) used initially in this study, all true variants except the *EGFR* p.S752_I759delSPKANKEI in CDX-268 specimen were detected. This variant was flagged as a “No-Call,” due to strong strand bias. As part of our policy, any “No-call” data for deleterious or actionable mutations is assessed on IGV and will be confirmed by an appropriate method.

As shown in Table 3, overall, based on the sequencing results from 35 samples initially used and evaluation of 134 indel and 2,178 SNV data points, the analytical sensitivity within the reportable range of the assay was defined to be >99% for SNVs, >99% for indels, and 99.7% for both combined (Detailed information can be find in the Table S9-**“SNVs and Indels tab”**).

**Table 3:** The overall analytical Sensitivity of the OCA v1 assay for the detection of SNVs, Indels, Fusion transcripts, and CNVs.

**Variant Type**	**Analytical Sensitivity (%)**
SNVs within the reportable range	99.77
Indels within the reportable range	99.29
Fusion transcripts	100.00
CNV≥8, tumor fraction ≥50%	96.15
CNV≥3, tumor fraction ≥50%	76.32
Homozygous deletion: CN = 0, tumor fraction ≥50%	100.00

To determine the analytical sensitivity of the assay in detecting CNAs, samples with various known CNAs were processed, and the data were analyzed in IR5.0 with a custom FFPE CNV baseline. For samples with ≥50% tumor cellularity, the analytical sensitivity of the assay for amplification of ≥8 was 96.15%, and the accuracy for detecting homozygous deletions (copy number, 0) was 100% (Table 3). Although the assay was able to detect the homozygous deletions of the covered genes with high accuracy, because tissue and tumor heterogeneity may have a significant impact on the percentage of DNA derived from tumor cells or different tumor sub-clones, the homozygous deletion detection of tumor suppressors was excluded from our reportable range. However, when this assay used for translational research purposes, the CN data was provided as research use only with a disclaimer emphasizing on the importance of confirming homozygous deletion or <7 copies variants by another confirmatory method. Detailed data used for calculating the analytical sensitivity of this assay for the detection of CNVs can be find in the Table S9-**“CNVs tab”**.

To evaluate the analytical sensitivity of the OCAv1 RNA fusion panel, six normal male samples (negative for ALK or ROS1 fusion), three clinical FFPE reference samples (confirmed by a third party for fusion events detected), 12 FFPE-mimic samples prepared from a cultured Vcap (TMPRSS2- ERG) cell line, a Horizon Discovery RNA multiplex ALK-ROS-RET reference standard, and three Horizon Discovery FISH reference standards were used. All fusions above the detection limit (1%) were detected (detailed data can be found in the Tables S9, S10). Therefore, the analytical sensitivity of the assay for fusion variant detection was defined to be 100% (Table 3).

### Reproducibility

To determine the reproducibility of the assay, three DNA libraries for Quantitative Multiplex FFPE reference standard and 3 RNA libraries for Horizon Discovery reference sample HD640 (2.5%, CCDC6-RET fusion) were prepared with different barcodes under the same conditions but run on different days by three different technologists. Based on the results, 100% agreement for all known variants in the Quantitative Multiplex FFPE reference standard above the LLOD and within the reportable range of the OCAv1 were achieved. The allele frequencies for four false-negative variants (*EGFR* p.L858R, 2235_2246del12, and p.T790M, and *NF2* p.P275fs) in the Quantitative Multiplex FFPE reference standard were lower than the LLOD of our assay. No false-positive calls were detected. The assay also reproducibly detected the *CCDC6-RET*12, *EML4-ALK*, and SLC34A-ROS1 fusions.

Two DNA libraries for a structural multiplex reference standard (HD753) were prepared and sequenced on different days by two different technologists under the same conditions but with different barcodes to assess the reproducibility of the CNA assay. The results showed 100% agreement for both the *MYCN* (9.5) and *MYC* (9.8) amplifications (CN > 8) present in this reference sample. The *MET* (4.5) amplification was below the LLOD and was not included in the analysis. The results of reproducibility studies were summarized in the Tables S11A–C.

### Specificity

The NIST RM8398 human DNA contains human genomic DNA extracted from a large growth of the human lymphoblastoid cell line GM12878 and is intended to provide a whole human genome sample and accompanying reference values to assess the performance of variant calling from genome sequencing. Specifically, the material can be used to obtain estimates of true positives, true negatives, and false negatives for variant calls. DNA libraries for RM8398 were prepared under the same conditions but run on two different runs to assess the specificity of the assay (Table S12).

A list of 135 true-positive variants (131 SNVs, 1 MNV, and three indels) within the reportable range of the OCAv1 was obtained by comparing the high-confidence variants in RM8398 with the bed file defining the region of interest in the assay. A total of 134 true positives were detected in one run, and 132 true positives were detected in the other run (Table S12-**“RM8398”**). There was one false-negative variant in both runs for the *NOTCH1* gene on chromosome nine at position 139410589 because of low coverage. No false-positive variant was detected in both runs. Based on these results, the analytical specificity of the assay for SNV and indel detection was 100% (Table S12-**“RM8398 summary”**).

The specificity of the CNA assay was determined to be 99.9% based on run data generated using the Horizon Discovery structural multiplex reference standard and 12 samples without any known CNAs (Table S12-**“CNA”**).

The specificity of the fusion assay was evaluated with three runs with the Horizon Dx FFPE FISH reference standards HD615 and eight samples that were known to be negative for any fusion variant (Table S12-**“fusion”**). One false-positive fusion event (CD74-ROS1, with 28 fusion transcript reads) was detected in one of the runs. The analytical specificity of the fusion assay was calculated to be 99.96%.

### Interference

In addition to the false positive calls in the background of a sequencing run, nucleic acid impurities in a sample can have an effect on the uniformity of coverage. Ethanol is used in several wash steps in various nucleic acid extraction processes. Particularly, in bead-based DNA extraction protocols ethanol carryover may occur if a testing personnel or an unadjusted liquid handler does not remove the washing solution completely. To define the effects of nucleic acid impurities on the quality of a sequencing run, different amounts of ethanol were added to the NIST DNA sample RM8398, the *ERBB2* amplification DNA reference standard, the FISH reference standard HD615, and the RNA multiplex ALK-ROS-RET reference standard. The interference runs would show whether remnants of ethanol interfered with library preparation, emulsion PCR and the sequencing reaction.

For the DNA panel, the amount of library generated was significantly decreased with an increase in ethanol concentration from 1 to 5%. At 10 and 20% ethanol concentrations, significant PCR inhibition was observed. For the RNA panel, ethanol interference led to lower library concentrations, whereas, with 10 and 20% ethanol contamination, the concentration of libraries were lower than the acceptable threshold (Table S13).

The interference runs showed that ethanol decreased the amount of amplified library produced. Although the percentage of mapped reads and the mean depth were only slightly affected by 1 and 5% ethanol in the DNA samples, the uniformity of coverage and the sensitivity of the assay decreased greatly. For the RM8398 1% ethanol sample, three SNVs (in the *DNMT3A, BRCA2*, and *NF1* genes) and one indel variant (in the *FLT3* gene) were not detected. An additional six false-positive calls were observed with low-quality reads, and >40% of the true variants were not detected in the RM8398 sample containing 5% ethanol. The purified samples showed very high-quality reads for all detected variants.

In regard to the fusion panel, only three out of four true fusion variants were detected, when 5% ethanol added into the RNA multiplex ALK-ROS-RET reference standard. This may be explained by the observation that the fusion read counts were decreased in samples containing 5% ethanol.

The interference results indicate that impurities in samples may negatively affect library construction and sequencing data. Ethanol carryover should be avoided during the DNA extraction process, but note that residual ethanol cannot be detected by the 260:280 or 260:230 ratios if extracted nucleic acids are eluted in Tris-HCl buffer.

### Performance of the OCAv1 Assay on FFPE and OEFF Specimens

DNA and RNA library preparation and sequencing were successful for 93% FFPE specimens (25 out of 27). For two specimens, there was not enough tissues for successful DNA extraction and library preparation. The 25 DNA libraries from various tumor types were sequenced with an average mapped read of 4,055,010 (97.05% on-target), an average depth of coverage of 1,623, and an average reads per amplicon of 1,557. The 27 RNA libraries were sequenced with an average mapped read of 613,642 (98.60% on-target) (Table S3), which is well-beyond the required total mapped fusion panel reads of 20,000, recommended by the manufacture. The uniformity of coverage for three samples were lower than 90% but higher than 80%; the target base coverage at 100× for these cases were higher than 94.5%. The results for these samples were evaluated in IGV, before final approval.

Previously, in one of our studies involving analyses of 17 prostate and 24 renal cell carcinoma (RCC) FFPE specimens with the Cancer Hotspot Panel v2 (CHPv2, ThermoFisher, CA) NGS assay, DNA extraction and library preparation were successful for 12 of the 17 (70.6%) prostate samples and 7 of the 24 (28%) RCC samples. Similar to what was described in this study, all five failed prostate samples had low DNA yield (2–28 pg/μL in 30 μL elution volume). Of the 17 failed RCC samples, 16 had low DNA yield (0–167 pg/μL in 30 μL elution volume). In all but 1 RCC case, PCR was successful when sufficient starting materials were used (20 ng DNA and 10 ng RNA for the OCAv1, and 10 ng DNA for the CHPv2). These results indicate that the amount of functional DNA that can be obtained is one of the limiting factors for testing old FFPE samples, due to DNA fragmentation or deamination process.

The amplicon size range for the CHPv2 was from 111 to 187 bp, with mean average size of 154 bp (ThermoFisher, CA). The average designed amplicon size of the OCAv1 is only 103 bp. Therefore, compared with that of the OCAv1 assay, the higher failure rate of the CHPv2 panel NGS assay on FFPE samples was also likely due to the larger amplicon sizes.

As part of this study, we also tested OCT-embedded fresh frozen blocks from 26 patients with seven different tumor types using the OCAv1. The 26 DNA libraries were sequenced with an average mapped read of 3,959,285 (97.40% on-target), mean depth of coverage of 1,603, reads per amplicon of 1511, 98.40% of targeted based covered by at least 100 reads, and 148 called variants per sample. The corresponding RNA libraries from the same samples were also sequenced with an average mapped read of 582,866 (95.06% on-target) (Table S3). Overall, as shown in Table 4, the difference between total mapped reads, uniformity of coverage, and average read per amplicon between FFPE and frozen specimen groups were not statistically significant based on Welch's *t*-test analysis, and the *P*-values for these metrics were 0.6, 0.15, and 0.5, respectively.

**Table 4:** Comparison of OCAv1 assay performances on OCT-embedded fresh frozen and FFPE specimens.

**Welch's** ***t*****-Test Results: FFPE vs. Frozen Samples**						
	**Mapped reads**		**Uniformity of base coverage**		**Average reads per amplicon**	
	**FFPE**	**Frozen**	**FFPE**	**Frozen**	**FFPE**	**Frozen**
Mean	4,055,010	3,959,285	0.93674	0.94864	1556.68	1511.342
95% CI	[−267765.8, 459216.3]		[−0.0282, 0.0045]		[−94.13856, 184.81395]	
*P*	0.5987		0.1491		0.5165	

## Molecular Signatures of OEFF Specimens Tested by OCAv1

**Figure 3:**
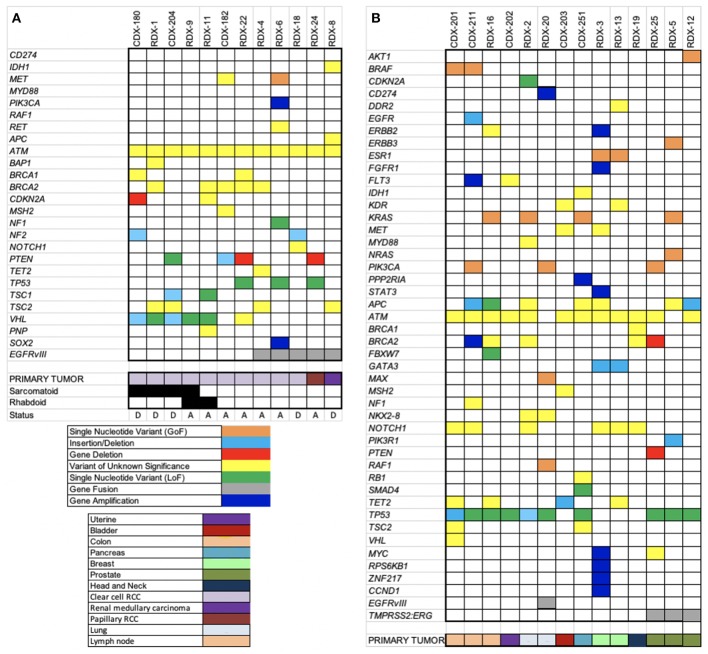
Heatmaps of somatic alterations detected. OCAv1 identified relevant somatic alterations in the 26 solid tumor registry specimens, including cases with RCC **(A)** and other types of cancers **(B)**. All relevant high-priority molecular alterations, including SNVs, indels, CNAs, fusions, and variants of unknown significance, are shown in the heat map. Specific alterations and cancer types are shown at the bottom of the heat maps according to the keys.

The performance of OCAv1 OEFF specimens was explained in the last section. After removing synonymous and non-coding variants detected, the alterations with minor allele frequency <2% were considered high-priority alterations for the variant annotation step. Based on these filtering criteria, an average of 5 SNVs, 0.6 indels, 0.7 CNAs, and 0.3 fusions were detected per sample (Table S14). Overall, 23 (88%) of these 26 patients had at least one loss-of-function or gain-of-function molecular alteration (Table S14 and Figures 3A,B).

In this study, a total of eight different missense SNVs in *ATM* gene were detected in all but one sample (Table S14), accounting for 22% of all the SNVs detected, and all of them were classified variants of unknown significance. The primary tumor for 12 cases enrolled in this study was RCC: 10 clear cell (cRCC), one papillary, and one medullary. Of the 10 patients with cRCC, six developed sarcomatoid and and/or rhabdoid features, defined by immunohistochemical and morphological evaluations of the submitted biopsies. The saromatoid variant of RCC is usually associated with poor prognosis and higher proliferative index (Bostrom et al., 2012). Based on histopathological review, case RDX-9 showed both sarcomatoid (only 5%) and rhabdoid features. Besides *ATM*, the most frequently altered gene in the cRCC specimens was *VHL* (60% of cRCC samples). Interestingly, in this study, only cases with sarcomatoid and/or rhabdoid features harbors loss-of-function alterations in *VHL* gene, most likely originating from the original carcinomatous lesion. Although it has been reported that mutations in *TP53* genes are more common in sarcomatoid tumors, no mutation in the *TP53* gene was detected in tumors with sarcomatoid or rhabdoid component. Molina et al. showed the overexpression of known markers linked to the mTOR activation pathway (p4E-BP1 and P-S6K) in the majority of cRCC with sarcomatoid variant that were included in their study (Molina et al., 2011). In concordance with the Molina et al. study, 3 of 5 sarcomatoid/rhabdoid variant-cRCC samples tested in our study were positive for deficient *TSC1, PTEN*, and *CDKN2A* genes. The *CDKN2A* gene encodes the P16(INK4A) and p14(ARF) proteins, both of which act as tumor suppressors. The P16(INK4A) is a cell cycle checkpoint inhibitor and p14(ARF) protects p53 from ubiquitin-dependent protein degradation. Therefore, both proteins inhibit cell growth and proliferation (Rivandi et al., 2017). PTEN and TSC1 proteins are also tumor suppressors that are involved in regulating the PI3K-AKT-mTOR pathway (Hermida et al., 2017; Nathan et al., 2017). It has been suggested that detection of mTOR activation, through expression analyses of markers such as pAKT, PTEN, pS6k, and p27 (CDKN1B) may be predictor of poor prognosis and respond to mTOR inhibitors. Figure 3A depicts all loss-of-function (LOF), gain-of-function (GOF), and unknown variants detected in RCC cohort. As shown in this figure, patient RDX-1 harbored a variant with unknown significance (p.Y173C) in the *BAP1* gene. This variant has been predicated to be an inactivating alteration (Klebe et al., 2015). However, because it has not been functionally characterized, we are reporting it as a variant of unknown significance. It has been reported that the *BAP1* gene is mutated in 12% of cRCC patients (Riazalhosseini and Lathrop, 2016).

As shown in Figure 3A, 5 RCC patients were also positive for *EGFRvIII* alteration; the primary tumor for 3 cases was cRCC (3 of 12 cRCC samples). The presence of the *EGFRvIII* mRNA was confirmed by TaqMan RT-PCR assay as well (Figure S1). However, the protein expression status of this variant was not assessed and verified. The presence of an *EGFRvIII* alteration in patients with RCC has not been reported previously to the best of our knowledge. A study by Kallio et al. (2003) reported that the overall survival was significantly higher in patients with RCC who had noticeable immunostaining for membranous EGFR (Kallio et al., 2003). However, this finding was challenged by Modjtahedi and Cunningham (2005), who stated that the antibody used for EGFR immunostaining in the Kallio et al. paper was a polyclonal rabbit anti-EGFR variant III antibody (Modjtahedi and Cunningham, 2005). To our knowledge, this issue has not been resolved.

The *EGFRvIII* alteration which harbors an in-frame deletion of 267 amino acids in the extracellular domain of EGFR protein leads to the lack of ligand binding domain and ligand-independent constitutive tyrosine kinase activity. This variant is common in EGFR-overexpressing gliomas. EGFRvIII protein activates or up-regulates several protein kinases and transcription factors involved in multiple signaling pathways in glioblastoma multiforme, including but not limited to STAT, PI3K-AKT-mTOR, and Ras-Raf-MAPK. In glioblastoma multiforme, STAT3 stimulates the up-regulation of VEGF (Chistiakov et al., 2017). These pathways are also important for cRCC pathogenesis. Previous studies using NGS have revealed the occurrence of evolutionary convergent phenotypic events, despite divergent genotypic alterations, in genes related to cRCC pathogenesis, including distinct mutations in genes involved in the PI3K-AKT-mTOR pathway, chromatin remodeling, and the VHL-HIF pathway (Voss et al., 2014; Riazalhosseini and Lathrop, 2016). Studies have also shown that the EGFR protein is overexpressed in both primary and metastatic RCC (Bayrak et al., 2014).

Considering these rationales, along with variant confirmation by qTR-PCR and the absence of false-positive *EGFRvIII* calls in the reference materials used in this study, together urged us to cautiously report this finding. However, we think the presence of this variant and its importance in RCC pathogenesis and prognosis should be evaluated in a larger cohort of samples.

## Discussion

In this study, the performance characteristics of a scalable and comprehensive targeted NGS pipeline, OCAv1, for detecting novel and known actionable somatic molecular alterations in solid tumors was assessed. Commercially available engineered tissues, FFPE-embedded cell lines, and previously tested FFPE specimens were used in this study. The performance of the assay was also evaluated on OEFF specimens (from UT solid tumor registry cohorts). Clinically, actionable variants detected in frozen samples were also confirmed by an appropriate orthogonal method.

The OCAv1 was designed to be an efficient, scalable, and cost-effective comprehensive NGS panel. This panel was designed based on genomic data from ~700,000 tumor specimens and frequent driver copy number and fusion alterations reported previously (Hovelson et al., 2015; Luthra et al., 2017). Although the panel covers only the hotspot mutations for a subset of the oncogenes, it assesses the entire coding sequences of the covered tumor suppressor genes. The analytical performance of this assay on PGM sequencer was first studied by Hovelson et al. (2015). Then Luthra et al. studied the analytical characteristics of the assay on Ion Proton (Luthra et al., 2017).

In our laboratory, we also validated the analytical performance of this panel for detection of SNVs, indels, fusion transcripts and CNAs on PGM sequencer using multiple sample types, non-cancer and cancerous FFPE tissues, OEFF biopsies, commercially available reference samples, and cell lines.

The Oncomine Comprehensive Cancer Panel requires relatively low amount of DNA and RNA. The overall performance of this test was acceptable: the sensitivity for detecting SNVs with allele frequencies >10% was >99%, that for known indels with allele frequencies >10% was >99%, that for indels (novel and known) with allele frequencies >20% was >95%, and that for high-level CNAs (>8 copies) was >95%. The tumor nuclei content was also determined to be a critical factor for detecting CNAs. Additionally, the specificity of the assay for detecting point mutations, CNAs, and fusion transcripts was found to be >99%. The high specificity of the assay is important, as it minimizes the number of variants that may need to be confirmed by an orthogonal method. Additionally, the aligned sequencing reads for positive or suspected variants (flagged by the variant caller software as “no calls”) are visually reviewed in IGV to minimize the rate of false-positive or false-negative calls. All known deleterious (predefined alterations) or likely deleterious alterations (with MAF <2%) are investigated further for actionability assessment and treatment prioritization or clinical trial matching.

Overall, the analytical validation of this molecular test shows highly concordant DNA and RNA sequencing results for reference materials and clinical specimens previously tested. This multiplexed amplification-based comprehensive panel provides a scalable and rapid tool for detecting all types of relevant alterations (SNVs, indels, CNAs, and known rearrangements) and most importantly is compatible with small and challenging tissue specimens.

Altogether, 53 tissue biopsies from 14 different cancer types such as renal, colon, uterine, bladder, pancreas, sarcoma, peritoneum, lung, breast, gallbladder, prostate, melanoma, liver, and hemangioprecytoma were successfully sequenced. The average target base coverage at 100× for the DNA panel was 98.2% and the minimum was 93.37%. The average uniformity of coverage for the DNA panel was 94.3%. The uniformity of coverage for three FFPE samples was lower than 90%, but higher than 80%. These samples were flagged, and their sequencing results were cautiously evaluated by examining the raw data. The average depth of coverage and the target base coverage at 100× for these three samples were higher >1,700 and >94.5%, respectively.

As shown for UT solid tumor registry's cohort of specimens, not only did this NGS assay detect at least one clinically actionable alteration, it can be a great translational research tool. For instance, using this panel, we detected *EGFRvIII* in significant numbers of RCC core biopsies (5 of 12 cRCC samples). The biological importance of the alteration in renal cancer growth and progression and its clinical importance as a therapeutic or prognostic marker needs to be investigated further.

Overall, the validation study for the comprehensive NGS system described here has shown that the Oncomine Comprehensive Cancer Panel is a robust and reliable assay and can be used routinely in diagnostic or clinical research laboratories.

## Data Availability

All datasets generated for this study are included in the manuscript/Supplementary Files.

## Ethics Statement

Tumor Specimens tested in this study were from informed consented patients enrolled in the IRB-approved clinical registry at The University of Texas Health Science Center at Houston–Oncology Division.

## Author Contributions

MD: conception and design, method development, analysis and interpretation of data, writing and revision of the manuscript, and study supervision. KR and RA: conception and design, analysis and interpretation of data, writing and revision of the manuscript, and study supervision. LL and MR: data acquisition, analysis, writing, and revision of the manuscript.

### Conflict of Interest Statement

KR was employed by the company Consultative Genomics, and by NX Prenatal, Inc. The remaining authors declare that the research was conducted in the absence of any commercial or financial relationships that could be construed as a potential conflict of interest.
