# Supplementary material for: Validation and Clinical Applications of a Comprehensive Next Generation Sequencing System for Molecular Characterization of Solid Cancer Tissues
Source: Front Mol Biosci. 2019 Sep 25;6:82. doi: 10.3389/fmolb.2019.00082 (PMC6798036; doi:10.3389/fmolb.2019.00082)
Supplement: Supplementary file 1 [file Data_Sheet_1.pdf]

Table S1A. Samples used for assessing the performance of OCP assay for detection of SNVs and Indels

Validation	Number of Samples	Sample Name	Sample Type
**LLOD**	6	Hotspot ladder 6 (47.9%)	FFPE
		Hotspot ladder 5 (29.46%)	FFPE
		Hotspot ladder 4 (18.44%)	FFPE
		Hotspot ladder 3 (11.04%)	FFPE
		Hotspot ladder 2 (5.36%)	FFPE
		Hotspot ladder 1 (2.75%)	FFPE
**Analytical sensitivity**	31	B903060	FFPE
		B903061	FFPE
		B903062	FFPE
		B903063	FFPE
		B903067	FFPE
		MultiMix A	FFPE
		MultiMix B	FFPE
		MultiMix C	FFPE
		MultiMix D	FFPE
		MultiMix E	FFPE
		MultiMix F	FFPE
		MultiMix G	FFPE
		MultiMix H	FFPE
		Quantitative Multiplex	FFPE
		Cancer Hotspot Control	FFPE
		EGFR Multiplex	FFPE
		CDX211	frozen
		CDX204	frozen
		CDX248	FFPE
		CDX249	FFPE
		CDX256	FFPE
		CDX268	FFPE
		CDX270	FFPE
		CDX281	FFPE
		CDX318	FFPE
		CDX323	FFPE
		CDX251	frozen
		CDX326	FFPE
		RM8398	DNA
		Hotspot Ladder 6 (47.9%)	FFPE
		Hotspot Ladder 5 (29.46%)	FFPE
**reproducibility**	1	QM	FFPE
**Interference**	1	RM8398 with 20% EtOH	DNA
		RM8398 with 10% EtOH	DNA
		RM8398 with 5% EtOH	DNA
		RM8398 with 1% EtOH	DNA
		RM8398	DNA
**Specificity**	1	RM8398	DNA

Table S1B. Samples used for assessing the performance of OCP assay for detection of fusions

Validation	Number of Samples	Sample Name	Sample Type
LLOD and Analytical sensitivity	44	B903061	FFPE
		B903062	FFPE
		B903063	FFPE
		B903064	FFPE
		B903065	FFPE
		B903067	FFPE
		HD231 (50%)	FFPE
		HD231 (20%)	FFPE
		HD231 (10%)	FFPE
		HD231 (5%)	FFPE
		HD231 (2.5%)	FFPE
		HD231 (1%)	FFPE
		HD640 (22%)	FFPE
		HD640 (10%)	FFPE
		HD640 (5%)	FFPE
		HD640 (2.5%)	FFPE
		HD640 (1%)	FFPE
		HD615 (63%)	FFPE
		HD615 (45%)	FFPE
		HD615 (30%)	FFPE
		HD615 (10%)	FFPE
		HD615 (5%)	FFPE
		HD615 (2.5%)	FFPE
		CDX256	FFPE
		CDX259	FFPE
		CDX318	FFPE
		Vcap (100%)	FFPE
		Vcap (80%)	FFPE
		Vcap (70%)	FFPE
		Vcap (60%)	FFPE
		Vcap (50%)	FFPE
		Vcap (40%)	FFPE
		Vcap (30%)	FFPE
		Vcap (15%)	FFPE
		Vcap (10%)	FFPE
		Vcap (5%)	FFPE
		Vcap (2.5%)	FFPE
		Vcap (1%)	FFPE
		HDx RNA multiplex 100%	FFPE
		HDx RNA multiplex 80%	FFPE
		HDx RNA multiplex 60%	FFPE
		HDx RNA multiplex 30%	FFPE
		HDx RNA multiplex 15%	FFPE
		HDx RNA multiplex 5%	FFPE
reproducibility	2	HD640 (2.5%)	FFPE
		HDx RNA multiplex 15%	FFPE
Interference	2	HD615 (63%) 20% EtOH	FFPE
		HD615 (63%) 10% EtOH	FFPE
		HD615 (63%) 5% EtOH	FFPE
		HD615 (63%) 1% EtOH	FFPE
		HD615 (63%)	FFPE
		RNA multiplex 20% EtOH	FFPE
		RNA multiplex 10% EtOH	FFPE
		RNA multiplex 5% EtOH	FFPE
		RNA multiplex 1% EtOH	FFPE
		RNA multiplex	FFPE
Specificity	9	HD615 (63%)	FFPE
		B903061	FFPE
		B903062	FFPE
		B903063	FFPE
		B903064	FFPE
		B903065	FFPE
		B903067	FFPE
		HCC1143BL 100% DNA	FFPE
		HCC2218BL 100% DNA	FFPE

Table 1C. Samples used for assessing the performance of OCP assay for detection of CNVs

Validation	Number of Samples/ CNVs	Sample Name	Sample Type	Expected CNVs
**LLOD**	2 samples/6 CNVs	HCC1143/ HCC1143BL 100%/0%	FFPE	CCND1(12), MYC (8), AKT1(11) and MDM2(9)
		HCC1143/ HCC1143BL 80%/20%	FFPE	CCND1(12), MYC (8), AKT1(11) and MDM2(9)
		HCC1143/ HCC1143BL 50%/50%	FFPE	CCND1(12), MYC (8), AKT1(11) and MDM2(9)
		HCC1143/ HCC1143BL 40%/60%	FFPE	CCND1(12), MYC (8), AKT1(11) and MDM2(9)
		HCC1143/ HCC1143BL 30%/70%	FFPE	CCND1(12), MYC (8), AKT1(11) and MDM2(9)
		HCC1143/ HCC1143BL 20%/80%	FFPE	CCND1(12), MYC (8), AKT1(11) and MDM2(9)
		HCC2218/HCC2218BL 100%/0%	FFPE	ERBB2 (9), CDH1 (0)
		HCC2218/HCC2218BL 80%/20%	FFPE	ERBB2 (9), CDH1 (0)
		HCC2218/HCC2218BL 50%/50%	FFPE	ERBB2 (9), CDH1 (0)
		HCC2218/HCC2218BL 40%/60%	FFPE	ERBB2 (9), CDH1 (0)
		HCC2218/HCC2218BL 30%/70%	FFPE	ERBB2 (9), CDH1 (0)
		HCC2218/HCC2218BL 20%/80%	FFPE	ERBB2 (9), CDH1 (0)
**Analytical sensitivity**	36	B903060	FFPE	Negative
		B903061	FFPE	Negative
		B903062	FFPE	Negative
		B903063	FFPE	Negative
		B903064	FFPE	Negative
		B903065	FFPE	Negative
		B903067	FFPE	Negative
		B903069	FFPE	Negative
		HCC1143 100%	fresh cell	CCND1(12), MYC (8), AKT1(11) and MDM2(9)
		HCC2218 100%	fresh cell	ERBB2 (9), CDH1 (0)
		NA10985 100%	fresh cell	VHL(1)
		HCC1143/ HCC1143BL 100%/0%	FFPE	CCND1(12), MYC (8), AKT1(11) and MDM2(9)
		HCC1143/ HCC1143BL 80%/20%	FFPE	CCND1(12), MYC (8), AKT1(11) and MDM2(9)
		HCC1143/ HCC1143BL 50%/50%	FFPE	CCND1(12), MYC (8), AKT1(11) and MDM2(9)
		HCC1143/ HCC1143BL 40%/60%	FFPE	CCND1(12), MYC (8), AKT1(11) and MDM2(9)
		HCC1143/ HCC1143BL 30%/70%	FFPE	CCND1(12), MYC (8), AKT1(11) and MDM2(9)
		HCC1143/ HCC1143BL 20%/80%	FFPE	CCND1(12), MYC (8), AKT1(11) and MDM2(9)
		HCC2218/HCC2218BL 100%/0%	FFPE	ERBB2 (9), CDH1 (0)
		HCC2218/HCC2218BL 80%/20%	FFPE	ERBB2 (9), CDH1 (0)
		HCC2218/HCC2218BL 50%/50%	FFPE	ERBB2 (9), CDH1 (0)
		HCC2218/HCC2218BL 40%/60%	FFPE	ERBB2 (9), CDH1 (0)
		HCC2218/HCC2218BL 30%/70%	FFPE	ERBB2 (9), CDH1 (0)
		HCC2218/HCC2218BL 20%/80%	FFPE	ERBB2 (9), CDH1 (0)
		H1770/BL1770 50%/50% DNA	FFPE	MYCN (13)
		H1395/BL1395 50%/50% DNA	FFPE	MCL1(10), MYC(10)

Validation	Number of Samples/ CNVs	Sample Name	Sample Type	Expected CNVs
		NA05067/NA09216 50%/50% DNA	FFPE	CD274 (3), PDCD1LG2(3), CDKN2A (3), MYCN(1)
		NA14164/NA10985 50%/50% DNA	FFPE	RB1(1), VHL (1)
		NA12606/NA14485 50%/50% DNA	FFPE	FLT3(3), BRCA2(3), RB1(3), FGFR1(3)
		BL1395 100% DNA	FFPE	Negative
		BL1770 100% DNA	FFPE	Negative
		NA20022/NA24385 50%/50% DNA	FFPE	PIK3CA (3), SOX2(3), ATP11B (3), DCUN1D1 (3)
		HCC1143BL 100% DNA	fresh cell	negative
		HCC2218BL 100% DNA	fresh cell	negatvive
		NA10985 100%	FFPE	VHL(1)
		HDx ERBB2 amplification FISH reference standard (HD-C511) DNA	DNA	ERBB2 (16)
		HDx structural multiplex reference standard (HD753) DNA	DNA	MET (4.5), MYCN (9.5), MYC (9.8)
**reproducibility**	1	HDx Structural multiplex reference standard (HD753) DNA	DNA	MET (4.5), MYCN (9.5), MYC (9.8)
**Interference**	1	HC-C511	DNA	ERBB2 (16)
		HC-C511 1% EtOH	DNA	ERBB2 (16)
		HC-C511 5% EtOH	DNA	ERBB2 (16)
		HC-C511 10% EtOH	DNA	ERBB2 (16)
		HC-C511 20% EtOH	DNA	ERBB2 (16)
**Specificity**	13	B903060	FFPE	Negative
		B903061	FFPE	Negative
		B903062	FFPE	Negative
		B903063	FFPE	Negative
		B903064	FFPE	Negative
		B903065	FFPE	Negative
		B903067	FFPE	Negative
		B903069	FFPE	Negative
		BL1395 100% DNA	FFPE	Negative
		BL1770 100% DNA	FFPE	Negative
		HCC1143BL 100% DNA	fresh cell	negative
		HCC2218BL 100% DNA	fresh cell	negatvive
		HDx Structural multiplex reference standard (HD753) DNA	DNA	MET (4.5), MYCN (9.5), MYC (9.8)
