# Supplementary material for: Validation and Clinical Applications of a Comprehensive Next Generation Sequencing System for Molecular Characterization of Solid Cancer Tissues
Source: Front Mol Biosci. 2019 Sep 25;6:82. doi: 10.3389/fmolb.2019.00082 (PMC6798036; doi:10.3389/fmolb.2019.00082)
Supplement: Supplementary file 2 [file Data_Sheet_2.pdf]

Table S2. Primers and Probes used in this study

| Target | Primer sequence | TaqMan probe sequence | Reference/Taqman Assay ID |
|---|---|---|---|
| ***EGFRvIII*** | Forward: CGGGCTCTGGAGGAAAAG | 6FAM-GTGACAGATCACGGCTCGT-MGBNFQ | PMID: 22323597 |
| | Reverse: AGGCCCTTCGCACTTCTTAC | | |
| ***TMPRSS2-ERG*_T2E4 (COSF28)** | N/A | N/A | Hs04396364_ft |
| **GAPDH** | N/A | N/A | Hs03929097_g1 |
