# Supplementary material for: Validation and Clinical Applications of a Comprehensive Next Generation Sequencing System for Molecular Characterization of Solid Cancer Tissues
Source: Front Mol Biosci. 2019 Sep 25;6:82. doi: 10.3389/fmolb.2019.00082 (PMC6798036; doi:10.3389/fmolb.2019.00082)
Supplement: Supplementary file 3 [file Data_Sheet_3.pdf]

Table S3A. Sequence Quality Data Metrics for Reference Samples

Run Name	Sample	Sample Type	DNA/RNA	Mapped reads	Mapped Reads On Target	Base Coverage (Mean Depth)	Uniformity of Base Coverage	Target base coverage at 100 X	Average reads per amplicon	Analysis included?
LOD 1-1	Hotspot ladder 6 (47.9%)	FFPE	DNA	4,217,329	97.96%	1,730	95.42%	99.10%	1,633	YES
	HD231 (50%)	FFPE	RNA	554,999	92.62%	-	-	-	-	YES
LOD 2-1	Hotspot ladder 5 (29.46%)	FFPE	DNA	4,285,494	98.31%	1,792	95.95%	98.66%	1,665	YES
	HD231 (20%)	FFPE	RNA	633,550	97.59%	-	-	-	-	YES
LOD 3-1	Hotspot ladder 4 (18.44%)	FFPE	DNA	4,393,469	98.29%	1,810	96.08%	98.64%	1,707	YES
	HD231 (10%)	FFPE	RNA	675,983	93.44%	-	-	-	-	YES
LOD 4-1	Hotspot ladder 3 (11.04%)	FFPE	DNA	3,879,093	98.29%	1,604	96.18%	98.58%	1,507	YES
	HD231 (5%)	FFPE	RNA	604,318	87.46%	-	-	-	-	YES
LOD 5-1	Hotspot ladder 2 (5.36%)	FFPE	DNA	4,113,694	97.96%	1,662	95.65%	98.57%	1,593	YES
	HD231 (2.5%)	FFPE	RNA	570,994	92.20%	-	-	-	-	YES
LOD 6-1	Hotspot ladder 1 (2.75%)	FFPE	DNA	4,841,574	98.00%	2,025	95.38%	98.53%	1,875	YES
	HD231 (1%)	FFPE	RNA	928,783	91.73%	-	-	-	-	YES
LOD 1-2	Hotspot ladder 6 (47.9%)	FFPE	DNA	3,911,460	97.67%	1,569	95.22%	98.97%	1,510	YES
	HD231 (50%)	FFPE	RNA	629,384	92.41%	-	-	-	-	YES
LOD 2-2	Hotspot ladder 5 (29.46%)	FFPE	DNA	4,076,379	98.25%	1,701	95.97%	98.48%	1,583	YES
	HD231 (20%)	FFPE	RNA	758,354	97.63%	-	-	-	-	YES
LOD 3-2	Hotspot ladder 4 (18.44%)	FFPE	DNA	3,917,689	98.08%	1,609	96.04%	98.56%	1,519	YES
	HD231 (10%)	FFPE	RNA	556,320	98.08%	-	-	-	-	YES
LOD 4-2	Hotspot ladder 3 (11.04%)	FFPE	DNA	4,437,680	97.99%	1,800	96.17%	98.64%	1,719	YES
	HD231 (5%)	FFPE	RNA	616,023	87.26%	-	-	-	-	YES
LOD 5-2	Hotspot ladder 2 (5.36%)	FFPE	DNA	4,378,090	98.10%	1,782	95.47%	98.68%	1,698	YES
	HD231 (2.5%)	FFPE	RNA	574,234	92.02%	-	-	-	-	YES
LOD 6-2	Hotspot ladder 1 (2.75%)	FFPE	DNA	3,878,493	97.58%	1,599	95.00%	98.43%	1,496	YES
	HD231 (1%)	FFPE	RNA	426,013	91.58%	-	-	-	-	YES
ACR-1	Biochain Normal Male B903060	FFPE	DNA	3,997,978	97.49%	1,551	96.32%	98.68%	1,541	YES
	Biochain Normal Male B903060	FFPE	RNA	76,797	37.26%	-	-	-	-	NO
ACR-2	Biochain Normal Male B903061	FFPE	DNA	3,946,570	97.94%	1,573	96.39%	98.46%	1,528	YES
	Biochain Normal Male B903061	FFPE	RNA	1,004,065	99.03%	-	-	-	-	YES
ACR-3	Biochain Normal Male B903062	FFPE	DNA	3,946,055	98.14%	1,544	95.51%	98.23%	1,531	YES
	Biochain Normal Male B903062	FFPE	RNA	735,082	99.51%	-	-	-	-	YES
ACR-4	Biochain Normal Male B903063	FFPE	DNA	3,669,185	96.85%	1,460	96.26%	98.24%	1,405	YES
	Biochain Normal Male B903063	FFPE	RNA	671,829	93.01%	-	-	-	-	YES
ACR-5	Biochain Normal Male B903064	FFPE	DNA	4,452,972	96.54%	1,742	96.73%	98.69%	1,699	YES
	Biochain Normal Male B903064	FFPE	RNA	981,926	98.90%	-	-	-	-	YES
ACR-6	Biochain Normal Male B903065	FFPE	DNA	3,943,645	97.18%	1,520	94.92%	98.05%	1,515	YES
	Biochain Normal Male B903065	FFPE	RNA	884,843	99.24%	-	-	-	-	YES
ACR-7	Biochain Normal Male B903066	FFPE	DNA	3,712,633	90.98%	1,271	83.06%	94.09%	1,335	NO
	Biochain Normal Male B903066	FFPE	RNA	1,463,182	96.79%	-	-	-	-	NO
ACR-8	Biochain Normal Male B903067	FFPE	DNA	3,598,199	97.79%	1,419	94.15%	97.73%	1,391	YES
	Biochain Normal Male B903067	FFPE	RNA	778,930	96.87%	-	-	-	-	YES
ACR-10	Biochain Normal Male B903069	FFPE	DNA	5,450,955	97.77%	2,143	96.13%	99.06%	2,107	YES
	Biochain Normal Male B903069	FFPE	RNA	292,265	89.24%	-	-	-	-	YES
ACR-11	AcroMetrix MultiMix A	FFPE	DNA	3,953,255	96.38%	1,585	95.17%	97.76%	1,506	YES
	HD640 (22%)	FFPE	RNA	466,194	99.06%	-	-	-	-	YES
ACR-12	AcroMetrix MultiMix B	FFPE	DNA	3,570,246	97.73%	1,449	88.00%	95.60%	1,379	YES
	HD640 (10%)	FFPE	RNA	344,563	98.06%	-	-	-	-	YES
ACR-13	AcroMetrix MultiMix C	FFPE	DNA	4,124,438	97.49%	1,624	87.52%	96.16%	1,589	YES
	HD640 (5%)	FFPE	RNA	687,061	96.91%	-	-	-	-	YES
ACR-14	AcroMetrix MultiMix D	FFPE	DNA	3,635,386	98.45%	1,451	89.83%	96.17%	1,415	YES
	HD640 (2.5%)	FFPE	RNA	930,019	95.61%	-	-	-	-	YES
ACR-15	AcroMetrix MultiMix E	FFPE	DNA	3,155,119	96.97%	1,271	91.93%	96.61%	1,209	YES
	HD640 (1%)	FFPE	RNA	1,277,689	94.86%	-	-	-	-	YES
ACR-16	AcroMetrix MultiMix F	FFPE	DNA	4,665,547	97.24%	1,915	89.55%	97.18%	1,793	YES
	HD615 (63%)	FFPE	RNA	1,158,840	98.80%	-	-	-	-	YES
ACR-17	AcroMetrix MultiMix G	FFPE	DNA	4,297,370	96.91%	1,765	95.45%	98.26%	1,646	YES
	HD615 (30%)	FFPE	RNA	576,743	97.42%	-	-	-	-	YES
ACR-18	AcroMetrix MultiMix H	FFPE	DNA	3,760,968	97.18%	1,532	96.22%	98.34%	1,445	YES
	HD615 (10%)	FFPE	RNA	506,674	98.18%	-	-	-	-	YES
ACR-20	AcroMetrix Cancer hotspot control	FFPE	DNA	5,306,272	90.15%	2,012	97.51%	99.24%	1,891	YES
	HD615 (5%)	FFPE	RNA	438,052	95.46%	-	-	-	-	YES
ACR-21	Horizon Dx EGFR multiplex	FFPE	DNA	4,153,896	93.99%	1,614	96.81%	98.77%	1,543	YES
	HD615 (2.5%)	FFPE	RNA	416,288	89.29%	-	-	-	-	YES
ACR-30	CDX143	FFPE	DNA	3,175,188	96.81%	1,248	97.35%	98.80%	1,215	YES
	CDX143	FFPE	RNA	564,819	98.61%	-	-	-	-	YES
ACR-31	CDX198	FFPE	DNA	3,674,808	96.10%	1,457	95.62%	98.12%	1,396	YES
	CDX198	FFPE	RNA	463,517	98.76%	-	-	-	-	YES
PRE-3	RM8398	Fresh cell culture DNA	DNA	4,208,342	97.50%	1,691	94.24%	98.09%	1,622	YES
	HD615 (63%)	FFPE	RNA	521,465	97.76%	-	-	-	-	YES
PRE-2/INF-5	RM8398	Fresh cell culture DNA	DNA	3,820,056	97.87%	1,552	95.10%	98.35%	1,478	YES
	HD615 (63%)	FFPE	RNA	742,233	98.47%	-	-	-	-	YES
RPD-1/ACR-19	QM	FFPE	DNA	4,142,843	97.70%	1,685	95.34%	99.11%	1,600	YES
	HD 640 (2.5%)	FFPE	RNA	793,602	96.83%	-	-	-	-	YES
RPD-2	QM	FFPE	DNA	4,186,482	97.18%	1,717	54.53%	67.05%	1,608	YES
	HD 640 (2.5%)	FFPE	RNA	742,897	94.98%	-	-	-	-	YES
RPD-3	QM	FFPE	DNA	3,141,854	97.33%	1,271	96.93%	98.69%	1,209	YES
	HD 640 (2.5%)	FFPE	RNA	1,471,997	94.57%	-	-	-	-	YES
INF-4	RM8398 1% EtOH	Fresh cell culture DNA	DNA	3,936,222	96.54%	1,573	90.81%	96.73%	1,502	YES
	HD615 (63%) 1% EtOH	FFPE	RNA	379,498	85.19%	-	-	-	-	YES
INF-3	RM8398 5% EtOH	Fresh cell culture DNA	DNA	3,765,704	92.72%	1,348	45.13%	50.72%	1,380	YES
	HD615 (63%) 5% EtOH	FFPE	RNA	284,391	85.61%	-	-	-	-	YES
INF-9	HC-C511	FFPE	DNA	4,428,736	93.53%	1,703	92.52%	98.19%	1,637	YES
	RNA multiplex	FFPE	RNA	60,945	97.67%	-	-	-	-	YES
INF-10	HC-C511 1% EtOH	FFPE	DNA	3,354,939	95.83%	1,365	92.26%	97.30%	1,271	YES
	RNA multiplex 1% EtOH	FFPE	RNA	524,348	99.14%	-	-	-	-	YES
INF-11	HC-C511 5% EtOH	FFPE	DNA	4,900,731	97.74%	1,993	81.62%	93.70%	1,893	YES
	RNA multiplex 5% EtOH	FFPE	RNA	225,102	99.10%	-	-	-	-	YES
INF-5 (on server)/ PRE-1	RM8398	Fresh cell culture DNA	DNA	3,509,986	96.01%	1,363	74.73%	88.11%	1,332	NO
	HD615 (63%)	FFPE	RNA	998,123	98.83%	-	-	-	-	YES
CNV-1-1	HCC1143/HCC1143BL 100%/0%	FFPE	DNA	3,717,448	97.67%	1,482	88.65%	96.69%	1,435	YES
	Vcap (100%)	FFPE	RNA	649,320	96.74%	-	-	-	-	YES
CNV-2-1	HCC1143/HCC1143BL 80%/20%	FFPE	DNA	4,132,148	97.06%	1,669	93.66%	98.04%	1,585	YES
	Vcap (80%)	FFPE	RNA	696,318	99.82%	-	-	-	-	YES
CNV-3-1	HCC1143/HCC1143BL 50%/50%	FFPE	DNA	4,836,655	95.37%	1,939	90.65%	97.56%	1,823	YES
	Vcap (70%)	FFPE	RNA	179,383	99.70%	-	-	-	-	YES
CNV-4-1	HCC1143/HCC1143BL 40%/60%	FFPE	DNA	5,920,683	95.07%	2,322	93.58%	98.63%	2,225	YES
	Vcap (60%)	FFPE	RNA	541,298	99.11%	-	-	-	-	YES
CNV-5-1	HCC1143/HCC1143BL 30%/70%	FFPE	DNA	5,050,711	96.91%	2,063	93.85%	98.29%	1,935	YES
	Vcap (50%)	FFPE	RNA	420,362	99.83%	-	-	-	-	YES
CNV-1-2	HCC1143/HCC1143BL 100%/0%	FFPE	DNA	3,156,598	97.67%	1,257	88.66%	96.22%	1,219	YES
	Vcap (100%)	FFPE	RNA	593,594	96.61%	-	-	-	-	YES
CNV-2-2	HCC1143/HCC1143BL 80%/20%	FFPE	DNA	3,735,232	97.11%	1,506	93.97%	97.94%	1,506	YES
	Vcap (80%)	FFPE	RNA	573,333	99.80%	-	-	-	-	YES
CNV-3-2	HCC1143/HCC1143BL 50%/50%	FFPE	DNA	3,891,456	94.98%	1,526	90.93%	97.00%	1,461	YES
	Vcap (70%)	FFPE	RNA	143,262	99.68%	-	-	-	-	YES
CNV-4-2	HCC1143/HCC1143BL 40%/60%	FFPE	DNA	3,784,924	95.14%	1,493	93.38%	97.84%	1,423	YES
	Vcap (60%)	FFPE	RNA	405,197	99.10%	-	-	-	-	YES
CNV-5-2	HCC1143/HCC1143BL 30%/70%	FFPE	DNA	5,188,374	94.06%	2,068	93.93%	98.33%	1,929	YES
	Vcap (50%)	FFPE	RNA	376,315	99.66%	-	-	-	-	YES
CNV-7-1	HCC2218/HCC1143BL 100%/0%	FFPE	DNA	3,536,593	95.94%	1,409	85.65%	96.33%	1,341	YES
	Vcap (30%)	FFPE	RNA	619,247	99.17%	-	-	-	-	YES
CNV-8-1	HCC2218/HCC2218BL 80%/20%	FFPE	DNA	3,768,655	93.85%	1,456	90.19%	97.65%	1,398	YES
	Vcap (15%)	FFPE	RNA	626,594	99.75%	-	-	-	-	YES
CNV-12-1//CNV-ACR-1	HCC2218/HCC2218BL 50%/50%	FFPE	DNA	4,227,347	97.48%	1,714	92.94%	98.61%	1,629	YES
	HD615 (45%)	FFPE	RNA	429,128	82.58%	-	-	-	-	YES
CNV-9-1	HCC2218/HCC2218BL 40%/60%	FFPE	DNA	4,054,962	96.27%	1,637	93.63%	98.29%	1,543	YES
	Vcap (5%)	FFPE	RNA	379,695	99.25%	-	-	-	-	YES
CNV-10-1	HCC2218/HCC2218BL 30%/70%	FFPE	DNA	4,015,812	95.16%	1,599	94.23%	98.13%	1,510	YES
	Vcap (2.5%)	FFPE	RNA	343,019	99.77%	-	-	-	-	YES
CNV-11-1	HCC2218/HCC2218BL 20%/80%	FFPE	DNA	3,370,824	93.76%	1,326	93.99%	97.69%	1,249	YES
	Vcap (1%)	FFPE	RNA	411,735	99.72%	-	-	-	-	YES
CNV-ACR-2	H1770/BL1770 50%/50% DNA	FFPE	DNA	5,725,366	97.62%	2,317	94.53%	98.85%	2,209	YES
CNV-ACR-3	H1395/BL1395 50%/50% DNA	FFPE	DNA	3,470,226	97.04%	1,374	89.75%	95.91%	1,331	YES
	Horizon Dx RNA multiplex 100%	FFPE	RNA	525,518	90.26%	-	-	-	-	YES
CNV-ACR-4	NA05067/NA09216 50%/50% DNA	FFPE	DNA	3,755,949	95.60%	1,495	94.34%	98.51%	1,419	YES
	Horizon Dx RNA multiplex 80%	FFPE	RNA	376,941	83.77%	-	-	-	-	YES
CNV-ACR-5	NA14164/NA10985 50%/50% DNA	FFPE	DNA	3,244,924	97.27%	1,312	93.83%	98.24%	1,248	YES
	Horizon Dx RNA multiplex 60%	FFPE	RNA	529,164	87.95%	-	-	-	-	YES
CNV-ACR-6	NA12606/NA14485 50%/50% DNA	FFPE	DNA	4,314,800	98.03%	1,757	94.59%	98.59%	1,672	YES
	Horizon Dx RNA multiplex 30%	FFPE	RNA	376,069	89.87%	-	-	-	-	YES
CNV-ACR-7	BL1395 100%	FFPE	DNA	5,867,636	96.06%	2,391	94.40%	98.35%	2,228	YES
CNV-ACR-8	BL1770 100%	FFPE	DNA	3,234,402	95.93%	1,290	95.56%	97.96%	1,226	YES
CNV-ACR-9	NA20022/NA24385 50%/50% DNA	FFPE	DNA	3,782,745	95.38%	1,482	94.71%	98.12%	1,426	YES
	CDX-259 RNA	FFPE	RNA	364054	0.9571	-	-	-	-	YES
CNV-ACR-10	HCC1143BL DNA	Fresh cell culture DNA	DNA	3,580,921	82.49%	1,245	97.42%	98.84%	1,168	YES
	HCC1143BL RNA	Fresh cell culture RNA	RNA	471,531	96.45%	-	-	-	-	YES
CNV-ACR-11	HCC2218BL 100% DNA	Fresh cell culture DNA	DNA	4,093,378	96.69%	1,655	96.99%	99.01%	1,564	YES
	HCC2218BL 100% RNA	Fresh cell culture RNA	RNA	379,091	99.27%	-	-	-	-	YES
CNV-ACR-12	NA10985 100%	FFPE	DNA	4,317,474	97.73%	1,771	93.09%	98.76%	1,668	YES
	CDX-348 RNA	FFPE	RNA	879984	0.9936	-	-	-	-	NO
RPD-4/CNV-ACR-14	Horizon Dx structural multiplex reference standard (HD753) DNA	DNA	DNA	3,503,521	97.47%	1,424	96.75%	98.88%	1,350	YES
	Horizon Dx RNA multiplex 15%	FFPE	RNA	239,959	83.09%	-	-	-	-	YES
RPD-5	Horizon Dx structural multiplex reference standard (HD753) DNA	DNA	DNA	3,243,918	95.08%	1,287	96.64%	98.63%	1,219	YES
	Horizon Dx RNA multiplex 15%	FFPE	RNA	684,565	95.58%	-	-	-	-	YES
CNV-ACR-15	Horizon Dx ERBB2 amplification FISH reference standard (HD-C511) DNA	DNA	DNA	4,473,925	97.93%	1,848	92.49%	98.53%	1,732	YES
	Horizon Dx RNA multiplex 5%	FFPE	RNA	1,032,664	87.56%	-	-	-	-	YES

Table S3B. Sequence Quality data metrics for patient samples sequenced

Sample	Age	Gender	Site	Cancer Type	Final Tumor cellularity	Sample Type	DNA/RNA	Mapped Reads	Mapped Reads on Target	Base Coverage (Mean Depth)	Uniformity of Base Coverage	Target base coverage at 100 X	Average Reads per Amplicon
CDX-180 Fz	58	F	Liver	RCC, metastasis	50%	Frozen	DNA	4,111,791	98.05%	1,668	94.31%	98.58%	1,594
CDX-180 Fz	58	F	Liver	RCC, metatasis	50%	Frozen	RNA	963,887	98.56%	-	-	-	-
CDX-182 Fz	57	F	Lymph node	RCC, metastasis	90%	Frozen	DNA	5,026,122	97.74%	2,071	96.87%	93.37%	1,588
CDX-182 Fz	57	F	Lymph node	RCC, metastasis	90%	Frozen	RNA	779,668	97.93%	-	-	-	-
CDX-201 Fz	67	F	Liver	Colon	70%	Frozen	DNA	4,640,322	97.55%	1,908	97.46%	99.41%	1,789
CDX-201 Fz	67	F	Liver	Colon	70%	Frozen	RNA	482,805	95.97%	-	-	-	-
CDX-202 Fz	63	F	Liver	Uterine	50%	Frozen	DNA	4,311,559	98.36%	1,759	91.36%	98.11%	1,676
CDX-202 Fz	63	F	Liver	Uterine	50%	Frozen	RNA	477,400	95.98%	-	-	-	-
CDX-203 Fz	86	M	Bladder	Bladder cancer	80%	Frozen	DNA	3,755,514	97.59%	1,508	94.83%	98.13%	1,449
CDX-203 Fz	86	M	Bladder	Bladder cancer	80%	Frozen	RNA	533,184	95.81%	-	-	-	-
CDX-204 Fz	69	M	Renal Fossa	RCC	80%	Frozen	DNA	3,979,239	98.20%	1,612	96.38%	99.02%	1,545
CDX-204 Fz	69	M	Renal Fossa	RCC	80%	Frozen	RNA	512,878	97.30%	-	-	-	-
CDX-211 Fz	69	M	Liver	Colorectal cancer with liver metastasis	80%	Frozen	DNA	3,532,912	97.36%	1,421	96.27%	99.16%	1,360
CDX-211 Fz	69	M	Liver	Colorectal cancer with liver metastasis	80%	Frozen	RNA	670,683	99.47%	-	-	-	-
CDX-248	61	M	Liver	Panceatic cancer with Liver metastasis	90%	FFPE	DNA	4,303,544	96.96%	1,749	95.65%	98.98%	1,649
CDX-248	61	M	Liver	Panceatic cancer with Liver metastasis	90%	FFPE	RNA	508,246	96.51%	-	-	-	-
CDX-249	67	F	Pancrease/ Liver	Panceatic cancer with Omentum metastasis	20%	FFPE	DNA	4,551,557	98.33%	1,847	93.94%	98.31%	1,769
CDX-249	67	F	Pancrease/ Liver	Panceatic cancer with Omentum metastasis	20%	FFPE	RNA	752,609	97.35%	-	-	-	-
CDX-251 Fz	65	F	Mesenteric nodule	Pancreatic	60%	Frozen	DNA	5,305,112	97.81%	2,190	95.58%	100.00%	2,051
CDX-251 Fz	65	F	Mesenteric nodule	Pancreatic	60%	Frozen	RNA	533,542	99.38%	-	-	-	-
CDX-254	79	M	R shoulder	High Grade Sarcoma	90%	FFPE	DNA	4,250,799	98.04%	1,726	83.40%	94.48%	1,647
CDX-254	79	M	R shoulder	High Grade Sarcoma	90%	FFPE	RNA	720,777	98.67%	-	-	-	-
CDX-255	56	F	Peritonium	Metastatic Adenocarcinoma	60%	FFPE	DNA	3,781,921	95.03%	1,476	93.28%	97.73%	1,421
CDX-255	56	F	Peritonium	Metastatic Adenocarcinoma	60%	FFPE	RNA	841,131	99.74%	-	-	-	-
CDX-256	62	F	R Plural Space	Adenocarcinoma of the Lung	70%	FFPE	DNA	3,705,878	93.20%	1,426	92.78%	98.21%	1,365
CDX-256	62	F	R Plural Space	Adenocarcinoma of the Lung	70%	FFPE	RNA	910,975	99.32%	-	-	-	-
CDX-259	74	M	Lung/Liver	Signet ring Mucinous carcinoma	90%	FFPE	DNA	4,209,459	95.38%	1,669	94.93%	98.23%	1,587
CDX-259	74	M	Lung/Liver	Signet ring Mucinous carcinoma	90%	FFPE	RNA	498,390	98.83%	-	-	-	-
CDX-268	42	F	R Lung	Non Small Cell Carcinoma	70%	FFPE	DNA	4,504,979	97.26%	1,759	87.66%	95.74%	1,732
CDX-268	42	F	R Lung	Non Small Cell Carcinoma	70%	FFPE	RNA	892,630	99.17%	-	-	-	-
CDX-270	75	F	L Pelvis	Poorly Differentiated carcinoma	70%	FFPE	DNA	4,088,631	97.51%	1,671	95.52%	99.07%	1,576
CDX-270	75	F	L Pelvis	Poorly Differentiated carcinoma	70%	FFPE	RNA	206,771	98.67%	-	-	-	-
CDX-274	63	M	Prostate	Adenocarcinoma	60%	FFPE	DNA	5,190,769	98.64%	2,056	83.87%	95.62%	2,024
CDX-274	63	M	Prostate	Adenocarcinoma	60%	FFPE	RNA	166,165	98.04%	-	-	-	-
CDX-280	65	F	Gallbladder	Invasive Adenocarcinoma	80%	FFPE	DNA	3,351,351	97.91%	1,326	94.08%	97.80%	1,297
CDX-280	65	F	Gallbladder	Invasive Adenocarcinoma	80%	FFPE	RNA	914,513	97.16%	-	-	-	-
CDX-281	69	M	Cecum	Invasive Adenocarcinoma	90%	FFPE	DNA	3,925,923	97.24%	1,551	94.96%	98.66%	1,509
CDX-281	69	M	Cecum	Invasive Adenocarcinoma	90%	FFPE	RNA	558,255	98.20%	-	-	-	-
CDX-287	67	F	R Colon	Metastatic Adenocarcinoma	80%	FFPE	DNA	3,689,410	97.77%	1,467	94.89%	98.51%	1,426
CDX-287	67	F	R Colon	Metastatic Adenocarcinoma	80%	FFPE	RNA	1,001,493	98.35%	-	-	-	-
CDX-304	49	F	L Parietal Occipital	Hemangiopericytoma	95%	FFPE	DNA	3,926,703	96.59%	1,624	95.90%	98.77%	1,499
CDX-304	49	F	L Parietal Occipital	Hemangiopericytoma	95%	FFPE	RNA	720,748	97.25%	-	-	-	-
CDX-310	75	M	Superficial Bladder wall	Papillary Transitional Cell Carcinoma (Bladder)	90%	FFPE	DNA	3,168,211	95.78%	1,214	92.60%	97.05%	1,199
CDX-310	75	M	Superficial Bladder wall	Papillary Transitional Cell Carcinoma (Bladder)	90%	FFPE	RNA	225,672	97.94%	-	-	-	-
CDX-311	51	F	Liver	Poorly Differentiated Metastatic Adenocarcinoma	60%	FFPE	DNA	4,696,329	97.09%	1,886	96.10%	98.89%	1,802
CDX-311	51	F	Liver	Poorly Differentiated Metastatic Adenocarcinoma	60%	FFPE	RNA	452,349	99.50%	-	-	-	-
CDX-317 RNA only	32	F	Liver	Metastatic Adenocarcinoma	60%	FFPE	RNA	376,460	99.63%	-	-	-	-
CDX-318	68	M	R Lung	Pulmonary Adnenocarcinoma	40%	FFPE	DNA	4,426,678	98.08%	1,802	95.24%	99.00%	1,716
CDX-318	68	M	R Lung	Pulmonary Adnenocarcinoma	40%	FFPE	RNA	356,544	99.54%	-	-	-	-
CDX-319	54	F	T4 Tumor	Hemangiopericytoma	100%	FFPE	DNA	4,588,626	97.33%	1,862	95.36%	99.03%	1,765
CDX-319	54	F	T4 Tumor	Hemangiopericytoma	100%	FFPE	RNA	599,149	99.35%	-	-	-	-
CDX-323	75	M	R. upper lobe of lung	Adnenocarcinoma	70%	FFPE	DNA	4,421,967	98.20%	1,793	93.68%	97.74%	1,716
CDX-323	75	M	R. upper lobe of lung	Adnenocarcinoma	70%	FFPE	RNA	646,464	98.39%	-	-	-	-
CDX-326	92	M	Left Axillary Lymph node	Metastatic Lung Adenocarcinoma	80%	FFPE	DNA	4,990,568	98.11%	2,061	95.32%	98.88%	1,935
CDX-326	92	M	Left Axillary Lymph node	Metastatic Lung Adenocarcinoma	80%	FFPE	RNA	966,987	99.42%	-	-	-	-
CDX-336 RNA only	78	M	R Lung	NSCLC/Poorly differentiated adenocarcinoma	80%	FFPE	RNA	276,198	99.19%	-	-	-	-
CDX-338	67	F	Distal Descending Colon	Moderatley Well Diiferentiated Invasive Adenocarcinoma	80%	FFPE	DNA	3,865,647	97.92%	1,543	93.39%	98.10%	1,496
CDX-338	67	F	Distal Descending Colon	Moderatley Well Diiferentiated Invasive Adenocarcinoma	80%	FFPE	RNA	888,923	97.83%	-	-	-	-
CDX-347	56	M	Rectum	Moderatley Diiferentiated Invasive Adenocarcinoma	90%	FFPE	DNA	3,898,937	97.47%	1,575	95.37%	98.29%	1,502
CDX-347	56	M	Rectum	Moderatley Diiferentiated Invasive Adenocarcinoma	90%	FFPE	RNA	927,150	99.14%	-	-	-	-
CDX-355	68	F	Uterus	Endometrial Adenocarcinoma	85%	FFPE	DNA	3,872,785	97.23%	1,542	95.63%	99.63%	1,488
CDX-355	68	F	Uterus	Endometrial Adenocarcinoma	85%	FFPE	RNA	658,738	99.01%	-	-	-	-
CDX-361	79	F	R Breast	Diffuse large B cell Lymphoma	90%	FFPE	DNA	3,114,581	96.32%	1,235	95.34%	98.76%	1,186
CDX-361	79	F	R Breast	Diffuse large B cell Lymphoma	90%	FFPE	RNA	472,663	98.61%	-	-	-	-
CDX143	NA	M				FFPE	DNA	3,175,188	96.81%	1,248	97.35%	98.80%	1,215
CDX143	NA	M				FFPE	RNA	564,819	98.61%	-	-	-	-
CDX198	NA	M	Lymph Nodes chest and L Neck	Metatatic Melanoma	95%	FFPE	DNA	3,674,808	96.10%	1,457	95.62%	98.12%	1,396
CDX198	NA	M	Lymph Nodes chest and L Neck	Metatatic Melanoma	95%	FFPE	RNA	463,517	98.76%	-	-	-	-
RDX-1	NA	F	Endobronchial lung bx	Met Clear Cell RCC	80%	Frozen	DNA	4,144,225	97.57%	1,675	90.14%	97.04%	1,598
RDX-1	NA	F	Endobronchial lung bx	Met Clear Cell RCC	80%	Frozen	RNA	791,942	98.63%	-	-	-	-
RDX-11	NA	M	L2 Vertebral Boody	RCC, Bone Metastasis	75%	Frozen	DNA	5,158,153	97.25%	2,094	96.24%	99.22%	1,983
RDX-11	NA	M	L2 Vertebral Boody	RCC, Bone Metastasis	75%	Frozen	RNA	172,353	97.13%	-	-	-	-
RDX-12	NA	M	Lung	Prostatic Adenocarcinoma	70%	Frozen	DNA	4,263,647	98.03%	1,735	96.01%	98.99%	1,652
RDX-12	NA	M	Lung	Prostatic Adenocarcinoma	70%	Frozen	RNA	338,449	99.06%	-	-	-	-
RDX-13	NA	F	R. Iliac Bone	Breast Carcinoma	10%	Frozen	DNA	3,916,327	97.46%	1,592	97.17%	99.15%	1,509
RDX-13	NA	F	R. Iliac Bone	Breast Carcinoma	10%	Frozen	RNA	660,515	96.83%	-	-	-	-
RDX-16	NA	NA	Liver core	CRC, Liver Met	70%	Frozen	DNA	5,325,118	97.31%	2,125	91.66%	98.88%	2,048
RDX-16	NA	NA	Liver core	CRC, Liver Met	70%	Frozen	RNA	146,842	98.24%	-	-	-	-
RDX-18	NA	M	R. Kidney	RCC	80%	Frozen	DNA	2,937,743	97.53%	1,194	96.18%	98.60%	1,132
RDX-18	NA	M	R. Kidney	RCC	80%	Frozen	RNA	1,585,391	97.71%	-	-	-	-
RDX-19	NA	NA	Right submandibular	Squamous Cell Carcinoma	50%	Frozen	DNA	4,117,819	96.90%	1,634	92.17%	98.17%	1,577
RDX-19	NA	NA	Right submandibular	Squamous Cell Carcinoma	50%	Frozen	RNA	290,991	99.20%	-	-	-	-
RDX-2	NA	F	TBNA-4R	RCC	60%	Frozen	DNA	3,447,419	96.93%	1,385	96.15%	99.13%	1,321
RDX-2	NA	F	TBNA-4R	RCC	60%	Frozen	RNA	393,494	97.62%	-	-	-	-
RDX-20	NA	M	Lung transbronchial	RCC	50%	Frozen	DNA	4,306,379	98.42%	1,780	94.22%	98.55%	1,675
RDX-20	NA	M	Lung transbronchial	RCC	50%	Frozen	RNA	813,505	98.62%	-	-	-	-
RDX-3	NA	F	Lymph node	Met Breast Carcinoma	70%	Frozen	DNA	3,492,407	96.07%	1,380	96.18%	98.70%	1,326
RDX-3	NA	F	Lymph node	Met Breast Carcinoma	70%	Frozen	RNA	629,077	98.84%	-	-	-	-
RDX-4	NA	M	Lung	Met, Clear Cell RCC	90%	Frozen	DNA	4,064,513	97.05%	1,647	95.12%	98.29%	1,559
RDX-4	NA	M	Lung	Met, Clear Cell RCC	90%	Frozen	RNA	918,031	99.02%	-	-	-	-
RDX-5	NA	M	Liver	Prostate	80%	Frozen	DNA	2,547,316	95.94%	987.7	96.11%	98.31%	965.9
RDX-5	NA	M	Liver	Prostate	80%	Frozen	RNA	84,710	70.14%	-	-	-	-
RDX-6	NA	M	11R Lymph Node	Clear Cell RCC	50%	Frozen	DNA	3,782,166	97.41%	1,541	94.68%	98.71%	1,456
RDX-6	NA	M	11R Lymph Node	Clear Cell RCC	50%	Frozen	RNA	748,392	84.21%	-	-	-	-
RDX-8	NA	M	Lung	Renal Collecting Duct Cell Carcinoma, Met	100%	Frozen	DNA	3,368,903	97.42%	1,346	92.86%	97.69%	1,297
RDX-8	NA	M	Lung	Renal Collecting Duct Cell Carcinoma, Met	100%	Frozen	RNA	371,926	98.50%	-	-	-	-
RDX-9	NA	M	R. Kidney	Clear Cell RCC	100%	Frozen	DNA	3,795,534	97.07%	1,543	96.00%	98.93%	1,456
RDX-9	NA	M	R. Kidney	Clear Cell RCC	100%	Frozen	RNA	135,610	64.69%	-	-	-	-
RDX-22	NA	M	Left Kidney	RCC	90%	Frozen	DNA	3,104,359	97.22%	1,262	96.39%	98.82%	1,193
RDX-22	NA	M	Left Kidney	RCC	90%	Frozen	RNA	621,200	96.22%	-	-	-	-
RDX-24	NA	M	TBNA subcranial LN	RCC	90%	Frozen	DNA	2,848,675	97.77%	1,130	91.73%	96.96%	1,101
RDX-24	NA	M	TBNA subcranial LN	RCC	90%	Frozen	RNA	813,980	97.42%				
RDX-25	NA	M	Lung	Prostate	90%	Frozen	DNA	3,658,129	96.38%	1,481	94.39%	98.54%	1,394
RDX-25	NA	M	Lung	Prostate	90%	Frozen	RNA	684,069	99.05%	-	-	-	-
