# Supplementary material for: Validation and Clinical Applications of a Comprehensive Next Generation Sequencing System for Molecular Characterization of Solid Cancer Tissues
Source: Front Mol Biosci. 2019 Sep 25;6:82. doi: 10.3389/fmolb.2019.00082 (PMC6798036; doi:10.3389/fmolb.2019.00082)
Supplement: Supplementary file 4 [file Data_Sheet_4.pdf]

Table S4. Minimal Average Coverage required for detecting SNVs and Indels

Locus	Type	Allele Coverage	Allele Frequency
chr9:133748414	SNV	131	5.34%
chr10:89692993	SNV	408	5.39%
chr10:89692980	SNV	407	5.41%
chr10:89690805	SNV	168	5.95%
chr9:139397768	SNV	302	5.96%
chr10:89692965	SNV	403	5.96%
chr3:10188200	SNV	441	6.35%
chr17:7577120	SNV	451	6.43%
chr9:133748391	SNV	137	6.57%
chr9:133748403	SNV	135	6.67%
chr16:68846137	SNV	317	6.94%
chr15:90631934	SNV	355	7.04%
chr9:133738349	SNV	451	7.10%
chr9:139399344	SNV	56	7.14%
chr9:139399350	SNV	56	7.14%
chr9:139399365	SNV	56	7.14%
chr10:43609942	INDEL (4 bp)	84	7.14%
chr4:55592178	INS (6 bp)	348	7.18%
chr10:43609096	SNV	277	7.22%
chr10:43609102	SNV	277	7.22%
chr9:133738342	SNV	449	7.35%
chr15:90631838	SNV	359	7.52%
chr9:133738357	SNV	451	7.54%
chr9:139399422	SNV	53	7.55%
chr9:133738363	SNV	448	7.59%
chr3:178952085	SNV	158	7.59%
chr4:1808331	SNV	246	7.72%
chr19:3118942	SNV	193	7.77%
chr4:55594221	SNV	178	7.87%
chr10:43613840	SNV	151	7.95%
chr15:66727455	SNV	113	7.96%
chr10:43617416	SNV	276	7.97%
chr10:43615568	MNV	199	8.04%

Locus	Type	Allele Coverage	Allele Frequency
chr17:37880220	SNV	869	8.06%
chr17:37880261	SNV	878	8.20%
chr13:28602329	SNV	473	8.25%
chr17:7574018	SNV	264	8.33%
chr17:7574026	SNV	262	8.40%
chr17:37880981	INS (12 bp)	332	8.43%
chr10:89692911	SNV	598	8.53%
chr11:534288	SNV	68	8.82%
chr9:5073770	SNV	237	8.86%
chr13:28610138	SNV	469	8.96%
chr10:89711972	INS (3 bp)	365	9.04%
chr16:68847282	SNV	429	9.09%
chr4:153247366	SNV	336	9.22%
chr4:55594258	SNV	173	9.25%
chr17:7577580	SNV	225	9.33%
chr11:108200958	SNV	182	9.34%
chr3:178952055	SNV	158	9.49%
chr4:55599284	SNV	179	9.50%
chr4:55144547	SNV	378	9.52%
chr10:89711992	SNV	367	9.54%
chr4:1806089	SNV	115	9.57%
chr4:1806153	SNV	114	9.65%
chr4:1806119	SNV	113	9.73%
chr17:7578235	SNV	226	9.73%
chr10:123274774	SNV	154	9.74%
chr10:123274794	SNV	154	9.74%
chr17:7577559	SNV	225	9.78%
chr12:25380283	SNV	414	9.90%
chr11:108218045	SNV	393	9.92%
chr17:7578203	SNV	241	9.96%
chr12:25380275	SNV	411	9.98%
chr4:55593661	SNV	519	10.02%
chr7:128850341	SNV	147	10.20%
chr17:7578196	SNV	232	10.34%
chr11:108206594	SNV	309	10.36%

Locus	Type	Allele Coverage	Allele Frequency
chr7:55248995	SNV	125	10.40%
chr3:178916944	SNV	143	10.49%
chr4:55592202	SNV	352	10.51%
chr17:7578190	SNV	233	10.73%
chr4:55144148	SNV	268	10.82%
chr11:533874	SNV	129	10.85%
chr10:89692830	SNV	239	10.88%
chr9:133747520	SNV	602	10.96%
chr11:108236087	SNV	591	11.00%
chr7:140453145	SNV	172	11.05%
chr7:140453154	SNV	172	11.05%
chr7:140453193	SNV	172	11.05%
chr3:178921553	SNV	425	11.06%
chr20:57484420	SNV	117	11.11%
chr2:29432664	SNV	242	11.16%
chr7:140453136	SNV	170	11.18%
chr3:178916936	SNV	143	11.19%
chr10:89692850	SNV	249	11.24%
chr9:133750319	SNV	245	11.43%
chr9:133750356	SNV	245	11.43%
chr3:10188245	SNV	314	11.46%
chr9:21971111	SNV	173	11.56%
chr2:209113112	SNV	466	11.59%
chr4:55152093	SNV	328	11.59%
chr4:153249384	SNV	275	11.64%
chr3:178916957	SNV	146	11.64%
chr3:10188210	SNV	309	11.65%
chr11:108170479	SNV	247	11.74%
chr11:108138003	SNV	577	11.79%
chr7:116423428	SNV	338	11.83%
chr7:55242464	DEL (15 bp)	253	11.86%
chr3:138665163	SNV	201	11.94%
chr7:55242452	SNV	259	11.97%
chr7:116423474	SNV	325	12.00%
chr12:112888189	SNV	691	12.01%

Locus	Type	Allele Coverage	Allele Frequency
chr12:112888165	SNV	695	12.09%
chr7:128846398	SNV	115	12.17%
chr12:112926908	SNV	402	12.19%
chr12:112888210	SNV	680	12.21%
chr10:123258034	SNV	384	12.24%
chr3:178936082	SNV	633	12.32%
chr17:37881332	SNV	582	12.37%
chr13:28592629	SNV	242	12.40%
chr13:28592642	SNV	242	12.40%
chr7:55242433	SNV	256	12.50%
chr12:112926888	SNV	399	12.53%
chr11:108205780	SNV	303	12.54%
chr7:55242427	SNV	255	12.55%
chr12:112926852	SNV	389	12.60%
chr12:112888199	SNV	688	12.65%
chr7:140481478	SNV	308	12.66%
chr7:128845101	SNV	347	12.68%
chr2:29443695	SNV	354	12.71%
chr3:41266101	MNV	118	12.71%
chr7:55233043	SNV	589	12.73%
chr11:108205769	SNV	306	12.75%
chr7:116339642	SNV	272	12.87%
chr3:178936074	SNV	613	12.89%
chr12:25398284	SNV	432	12.96%
chr1:115256529	SNV	207	13.04%
chr5:149433645	SNV	137	13.14%
chr3:178938860	SNV	743	13.19%
chr3:178936091	SNV	650	13.23%
chr1:115258747	SNV	211	13.27%
chr9:80412493	SNV	241	13.28%
chr3:178947827	SNV	670	13.28%
chr11:108117798	SNV	159	13.84%
chr19:1221319	SNV	581	13.94%
chr1:115258730	SNV	215	13.95%
chr1:43815020	SNV	150	14.00%

Locus	Type	Allele Coverage	Allele Frequency
chr11:108123551	SNV	299	14.05%
chr11:108119823	SNV	377	14.06%
chr4:55961023	SNV	434	14.29%
chr12:25378647	SNV	301	14.29%
chr1:43815009	SNV	153	14.38%
chr7:116340262	SNV	556	14.39%
chr7:55211080	SNV	222	14.41%
chr1:43814979	SNV	152	14.47%
chr7:55259554	SNV	310	14.52%
chr20:57484596	SNV	251	14.74%
chr11:108218089	SNV	255	14.90%
chr19:1223125	SNV	179	15.08%
chr9:21971017	SNV	78	15.38%
chr10:123279677	SNV	103	15.53%
chr7:148508727	SNV	410	16.10%
chr10:89685307	SNV	86	16.28%
chr7:55249131	SNV	278	16.91%
chr7:55221822	SNV	212	16.98%
chr9:21971036	SNV	75	17.33%
chr3:10191479	SNV	277	17.33%
chr3:10191506	SNV	277	17.33%
chr3:10191513	SNV	277	17.33%
chr7:55249143	SNV	278	17.63%
chr3:178927980	SNV	33	18.18%
