# Supplementary material for: Validation and Clinical Applications of a Comprehensive Next Generation Sequencing System for Molecular Characterization of Solid Cancer Tissues
Source: Front Mol Biosci. 2019 Sep 25;6:82. doi: 10.3389/fmolb.2019.00082 (PMC6798036; doi:10.3389/fmolb.2019.00082)
Supplement: Supplementary file 5 [file Data_Sheet_5.pdf]

Table S5. Limit of Detection study for detection of SNVs and Indels																
**Gene**	**Type**	**In Hotspot .Bed file**	**Variant ID**	**Protein**	**Ladder 6 (50%)**		**Ladder 5 (30%)**		**Ladder 4 (20%)**		**Ladder 3 (10%)**		**Ladder 2 (5%)**		**Ladder 1 (2.5%)**	
					**1-1**	**1-2**	**2-1**	**2-2**	**3-1**	**3-2**	**4-1**	**4-2**	**5-1**	**5-2**	**6-1**	**6-2**
HRAS	SNV		COSM249860	p.(=)												
KDR	SNV		COSM35855	p.(=)												
FGFR3	SNV		COSM719	p.Lys650Glu												
RB1	SNV		COSM13117	p.Q685*												
HRAS	SNV		COSM499	p.Gln61Arg												
HRAS	SNV		COSM495	p.Ala59Thr												
HRAS	SNV		COSM483	p.Gly12Val												
STK11	SNV		COSM21570													
STK11	SNV		COSM29005	p.(=)												
STK11	SNV		COSM21355	p.Pro281Leu												
STK11	SNV		COSM21360	p.Phe354Leu												
FGFR3	SNV		COSM716	p.Gly370Cys												
FGFR3	SNV		COSM24842	p.Gly380Arg												
FGFR3	SNV		COSM724	p.Phe384Leu												
FGFR3	SNV		COSM721	p.Ala391Glu												
FGFR3	SNV		COSM1428724	p.H643R												
FGFR3	SNV		COSM24802	p.Gly697Cys												
FGFR3	SNV		AMXsynt1	p.(=)												
GNA11	SNV		COSM52969	p.Gln209Leu												
JAK2	SNV		COSM12600	p.Val617Phe												
JAK2	SNV		COSM27063	p.Asp620Glu												
TP53	SNV		COSM307348	p.Q375*												
TP53	SNV		COSM1191161	p.?												
TP53	SNV		COSM11073	p.Arg342Ter												
TP53	SNV		COSM11286	p.Glu339Ter												
TP53	SNV		COSM11071	p.Arg337Cys												
TP53	SNV		COSM11514	p.Gly334Val												

TP53	SNV		COSM11354	p.Gln331Ter
TP53	SNV		COSM44823	p.Tyr327Ter
TP53	SNV		COSM46088	p.(=)
TP53	SNV		COSM10786	p.Gln317Ter
TP53	SNV		COSM10660	p.Arg273His
TP53	SNV		COSM10812	p.Ser241Phe
TP53	SNV		COSM10725	p.Tyr234Cys
TP53	SNV		COSM10758	p.Tyr220Cys
TP53	SNV		COSM44317	p.Val218Glu
TP53	SNV		COSM10667	p.Val216Met
TP53	SNV		COSM43947	p.Tyr205Cys
TP53	SNV		COSM12168	p.Glu56Ter
TP53	SNV		COSM44907	p.Glu51Ter
TP53	SNV		COSM43664	p.Leu45Pro
TP53	SNV		COSM46286	p.Gln38Ter
VHL	SNV		COSM17752	p.His115Asn
VHL	SNV		COSM14312	p.Leu118Pro
VHL	SNV		COSM14407	p.Val130Leu
VHL	SNV		COSM17657	p.Leu158Val
VHL	SNV		COSM17612	p.Arg161Ter
VHL	SNV		COSM14311	p.Arg167Trp
VHL	SNV		COSM17837	p.Leu169Pro
JAK3	SNV		COSM34213	p.Val722Ile
JAK3	SNV		COSM34214	p.Ala572Val
CDKN2A	SNV		COSM12479	p.Glu120Ter
CDKN2A	SNV		COSM12476	p.Pro114Leu
CDKN2A	SNV		COSM12547	p.Trp110Ter
CDKN2A	SNV		COSM13489	p.Asp108Tyr
CDKN2A	SNV		COSM12504	p.His83Tyr
CDKN2A	SNV		COSM12475	p.Arg80Ter
CDKN2A	SNV		COSM13281	p.Glu69Ter
CDKN2A	SNV		COSM12473	p.Arg58Ter
SMARCB1	SNV		COSM1002	p.Arg40Ter
SMARCB1	SNV		COSM991	p.Tyr47Ter
SMARCB1	SNV		COSM24595	p.Arg53Ter

SMARCB1	SNV		COSM992	p.Arg158Ter												
SMARCB1	SNV		COSM993	p.Arg201Ter												
SMARCB1	SNV		COSM999	p.Ala203Thr												
KRAS	SNV		COSM19940	p.Lys117Asn												
KRAS	SNV		COSM554	p.Gln61His												
KRAS	SNV		COSM546	p.Ala59Thr												
KRAS	SNV		AMXsynt11													
KRAS	SNV		COSM14208	p.Thr35Ile												
KRAS	SNV		COSM521	p.Gly12Asp												
KRAS	SNV		COSM507	p.(=)												
FLT3	SNV		COSM1166729	p.Asp839Gly												
FLT3	SNV		COSM783	p.Asp835Tyr												
FLT3	SNV		COSM25248	p.Gly831Glu												
FLT3	SNV		COSM786	p.Ala680Val												
FLT3	SNV		COSM19522	p.Val592Ala												
FLT3	SNV		COSM28042	p.Ser451Phe												
ALK	SNV		COSM28056	p.Arg1275Gln												
ALK	SNV		COSM28055	p.Phe1174Leu												
MLH1	SNV		COSM26085	p.Val384Asp												
ERBB2	SNV		COSM14060	p.Leu755Ser												
ERBB2	SNV		COSM1251412	p.Asp769Tyr												
ERBB2	SNV		COSM14065	p.Val842Ile												
ERBB2	SNV		COSM686	p.Asn857Ser												
ERBB2	SNV		COSM21985	p.His878Tyr												
FGFR1	SNV		COSM1292693	p.(=)												
FGFR1	SNV		COSM187237													
FGFR1	SNV		COSM1456955	p.Thr174Ala												
FGFR1	SNV		COSM601	p.Ser158Leu												
CTNNB1	SNV		COSM5677	p.Ser33Cys												
RET	SNV		COSM29803	p.Cys618Arg												
RET	SNV		COSM29804	p.Cys620Arg												
RET	SNV		COSM1223553	p.Val648Ile												
RET	SNV		COSM21338	p.Glu768Asp												
RET	SNV		COSM977	p.Ala883Phe												
RET	SNV		COSM963	p.Glu901Lys												

RET	SNV		COSM965	p.Met918Thr												
MPL	SNV		COSM27286	p.Ser505Asn												
MPL	SNV		COSM18918	p.Trp515Leu												
MPL	SNV		COSM27290	p.Ala519Thr												
SMAD4	SNV		COSM1389031	p.(=)												
SMAD4	SNV		COSM14229	p.Val126Ala												
SMAD4	SNV		COSM218557	p.Pro130Leu												
SMAD4	SNV		COSM14168	p.Arg135Ter												
SMAD4	SNV		COSM13115	p.Ser144Ter												
SMAD4	SNV		COSM14118	p.Gly168Ter												
SMAD4	SNV		COSM1226725	p.Ser178Ter												
SMAD4	SNV		COSM308153	p.Gln183Ter												
SMAD4	SNV		COSM14057	p.Gln245Ter												
SMAD4	SNV		COSM22901	p.Gln256Ter												
SMAD4	SNV		COSM14163	p.Gln311Ter												
SMAD4	SNV		COSM14167													
SMAD4	SNV		COSM1389054	p.Gln334Arg												
SMAD4	SNV		COSM1389057	p.Glu337Gly												
SMAD4	SNV		COSM14109	p.Lys340Glu												
SMAD4	SNV		COSM14111	p.Ser343Ter												
SMAD4	SNV		COSM14249	p.Gly386Arg												
SMAD4	SNV		COSM14103	p.Ala406Thr												
SMAD4	SNV		AMXsynt12	p.Arg416Gly												
SMAD4	SNV		COSM14096	p.Arg445Ter												
SMAD4	SNV		COSM14114	p.Arg502Gly												
SMAD4	SNV		COSM1389099	p.Lys507Glu												
SMAD4	SNV		COSM14134	p.Glu526Ter												
SMAD4	SNV		COSM1389106	p.(=)												
RB1	SNV		COSM890	p.Glu137Ter												
RB1	SNV		COSM915	p.Leu199Ter												
RB1	SNV		COSM28816													
RB1	SNV		COSM891	p.Arg320Ter												
RB1	SNV		COSM1367204	p.Glu323Gly												
RB1	SNV		COSM1367206	p.Asn328Asp												
RB1	SNV		COSM879	p.Arg358Ter												

RB1	SNV		COSM895	p.Arg455Ter
RB1	SNV		COSM888	p.Arg556Ter
RB1	SNV		COSM1367255	p.W563R
RB1	SNV		COSM892	p.Arg579Ter
RB1	SNV		COSM1042	
RB1	SNV		COSM883	p.Cys706Phe
RB1	SNV		COSM868	p.Glu748Ter
RB1	SNV		COSM916	p.Val754Gly
RB1	SNV		COSM551465	p.Tyr756Cys
RB1	SNV		COSM254910	p.Lys765Ter
PDGFRA	SNV		COSM1430085	p.P581P
PDGFRA	SNV		COSM22415	p.Asn659Lys
PDGFRA	SNV		COSM1430086	p.(=)
PDGFRA	SNV		COSM743	p.Thr674Ile
PDGFRA	SNV		COSM587613	p.(=)
PDGFRA	SNV		COSM736	p.Asp842Val
PDGFRA	SNV		COSM28052	p.Asn848Lys
EGFR	SNV		COSM21683	p.Arg108Lys
EGFR	SNV		COSM174732	p.Glu114Lys
EGFR	SNV		COSM21687	p.Ala289Val
EGFR	SNV		COSM43067	p.Val292Leu
EGFR	SNV		COSM21690	p.Gly598Val
EGFR	SNV		COSM53194	p.Pro733Ser
EGFR	SNV		COSM13182	p.Gly735Ser
EGFR	SNV		COSM17570	p.Pro741Leu
EGFR	SNV		COSM28603	p.Val765Met
EGFR	SNV		COSM13190	p.Leu792Pro
EGFR	SNV		COSM12986	p.Gly810Asp
EGFR	SNV		COSM28610	p.Leu814Pro
EGFR	SNV		COSM13008	p.Ala871Gly
KIT	SNV		COSM96867	p.Phe506Leu
KIT	SNV		COSM96885	p.Lys509Ile
KIT	SNV		COSM1430136	p.Asn512Ser
KIT	SNV		COSM1155	p.Val530Ile
KIT	SNV		COSM1290	p.Leu576Pro

KIT	SNV		COSM1299	p.(=)
KIT	SNV		COSM1304	p.Lys642Glu
KIT	SNV		COSM12706	p.Val654Ala
KIT	SNV		COSM1430171	p.(=)
KIT	SNV		COSM20402	p.Arg804Trp
KIT	SNV		COSM133767	p.Trp853Ter
KDR	SNV		COSM1430203	p.Gly1145Arg
KDR	SNV		COSM48464	p.Ala973Ser
KDR	SNV		COSM1430212	p.(=)
KDR	SNV		COSM32339	p.Arg275Leu
GNAS	SNV		COSM27887	p.Arg201Cys
GNAS	SNV		COSM27888	p.Gln227Leu
MAP2K1	SNV		COSM1235478	p.Lys57Asn
MAP2K1	SNV		COSM1235479	p.Asp67Asn
CDH1	SNV		COSM1379165	p.Glu353Gly
CDH1	SNV		COSM19748	p.Asp370His
CDH1	SNV		COSM19750	p.Asp402Asn
CDH1	SNV		COSM19743	p.Ala617Thr
CDH1	SNV		COSM19822	p.Ala634Val
CDH1	SNV		COSM19418	p.Trp638Ter
GNAQ	SNV		COSM52975	p.Arg183Gln
GNAQ	SNV		COSM1463119	p.Thr175Ser
PTEN	SNV		COSM5298	p.Glu7Ter
PTEN	SNV		COSM5257	p.Phe56Val
PTEN	SNV		COSM5036	p.Tyr68His
PTEN	SNV		COSM5916	
PTEN	SNV		COSM5102	p.Cys71Tyr
PTEN	SNV		COSM5205	p.Asn82Thr
PTEN	SNV		COSM5983	
PTEN	SNV		COSM5266	p.Cys105Phe
PTEN	SNV		COSM5199	p.Leu112Val
PTEN	SNV		COSM5123	p.Gly132Asp
PTEN	SNV		COSM5130	p.Glu150Gly
PTEN	SNV		COSM5144	p.Tyr155Cys
PTEN	SNV		COSM5287	p.Arg159Ser

PTEN	SNV		COSM35406	p.Leu193Pro
PTEN	SNV		COSM5279	p.Pro204Thr
PTEN	SNV		COSM5072	p.Met205Ile
PTEN	SNV		COSM35849	p.Phe241Leu
PTEN	SNV		COSM43075	p.Lys263Ter
PTEN	SNV		COSM1349606	p.(=)
PTEN	SNV		COSM5312	p.Glu299Ter
PTEN	SNV		COSM1349625	p.Lys342Arg
PTEN	SNV		COSM1349633	p.Glu352Gly
PTEN	SNV		COSM23645	p.Ser364Cys
IDH2	SNV		COSM33733	p.Arg172Lys
IDH2	SNV		COSM1375400	p.(=)
IDH2	SNV		COSM41590	p.Arg140Gln
AKT1	SNV		COSM33765	p.Glu17Lys
ATM	SNV		COSM21323	p.Arg337Cys
ATM	SNV		COSM21825	p.Val410Ala
ATM	SNV		COSM22499	p.Pro604Ser
ATM	SNV		COSM1158828	
ATM	SNV		COSM21826	p.Phe858Leu
ATM	SNV		COSM22507	p.Ala1309Thr
ATM	SNV		COSM21920	p.Asp1682Tyr
ATM	SNV		COSM218294	p.Leu1718Val
ATM	SNV		COSM49005	
ATM	SNV		COSM172204	p.Arg1730Ter
ATM	SNV		COSM21918	p.Ala1742Pro
ATM	SNV		COSM12792	p.(=)
ATM	SNV		COSM1183962	p.Leu1826Val
ATM	SNV		COSM21922	p.Val1941Leu
ATM	SNV		COSM12951	p.Gln2442Pro
ATM	SNV		COSM12791	p.Thr2666Ala
ATM	SNV		COSM21636	p.Gly2695Ala
ATM	SNV		COSM1235404	p.Pro2699Thr
ATM	SNV		COSM22481	p.Asp2725Val
ATM	SNV		COSM1183939	p.Asn2875Ser
ATM	SNV		COSM22485	p.Leu2890Val

ATM	SNV		COSM21930	p.Thr2947Ser												
ATM	SNV		COSM21626	p.Arg3008His												
ATM	SNV		COSM1351060	p.(=)												
ATM	SNV		COSM21624	p.Arg3047Ter												
APC	SNV		COSM18852	p.Arg876Ter												
APC	SNV		COSM19065	p.Glu918Ter												
APC	SNV		COSM1432250	p.Tyr1102Cys												
APC	SNV		COSM1432260	p.(=)												
APC	SNV		COSM19072	p.Gln1291Ter												
APC	SNV		COSM18960	p.Gln1294Ter												
APC	SNV		COSM19048	p.Glu1353Ter												
APC	SNV		COSM19652	p.Ser1355Pro												
APC	SNV		COSM18862	p.Gln1378Ter												
APC	SNV		COSM143913	p.Pro1381Ser												
APC	SNV		COSM19087	p.Gln1406Ter												
APC	SNV		COSM18836	p.Gln1429Ter												
APC	SNV		COSM13879	p.Glu1547Ter												
APC	SNV		COSM41616	p.Glu1552Ter												
APC	SNV		COSM42906	p.Pro1609Leu												
PTPN11	SNV		COSM13011	p.Asp61Tyr												
PTPN11	SNV		COSM13013	p.Glu69Lys												
PTPN11	SNV		COSM13015	p.Ala72Val												
PTPN11	SNV		COSM13000	p.Glu76Lys												
PTPN11	SNV		COSM13034	p.Pro491Leu												
PTPN11	SNV		COSM13027	p.Gly503Ala												
PTPN11	SNV		COSM1358900	p.Thr507Ala												
PTPN11	SNV		COSM13031	p.Gln510Lys												
NRAS	SNV		COSM584	p.Gln61Arg												
NRAS	SNV		COSM1332933	p.(=)												
NRAS	SNV		COSM577	p.Ala18Thr												
NRAS	SNV		COSM564	p.Gly12Asp												
NRAS	SNV		COSM24850	p.Gly10Glu												
MET	SNV		COSM706	p.Glu168Asp												
MET	SNV		COSM710	p.Asn375Ser												
MET	SNV		COSM697	p.His1124Asp												

MET	SNV		COSM700	p.Tyr1253Asp												
MET	SNV		COSM48565	p.Gly1260Cys												
MET	SNV		COSM695	p.Lys1262Arg												
MET	SNV		COSM691	p.Met1268Thr												
FGFR2	SNV		COSM36912	p.Asn549Lys												
FGFR2	SNV		COSM36906	p.Cys382Arg												
FGFR2	SNV		COSM36904	p.Tyr375Cys												
FGFR2	SNV		COSM1346272	p.Thr370Ala												
FGFR2	SNV		COSM36901	p.Lys310Arg												
FGFR2	SNV		COSM29824	p.Gly305Arg												
FGFR2	SNV		COSM36903	p.Ser252Trp												
SMO	SNV		COSM13145	p.Arg199Trp												
SMO	SNV		COSM13147	p.Ala324Thr												
SMO	SNV		COSM216037	p.Leu412Phe												
SMO	SNV		COSM13146	p.Trp535Leu												
SMO	SNV		COSM13150	p.Thr640Ala												
ABL1	SNV		COSM12631	p.Leu248Val												
ABL1	SNV		COSM12577	p.Gly250Glu												
ABL1	SNV		COSM12576	p.Tyr253His												
ABL1	SNV		COSM12573	p.Glu255Lys												
ABL1	SNV		COSM12602	p.Asp276Gly												
ABL1	SNV		COSM12578	p.Met351Thr												
ABL1	SNV		COSM12611	p.Glu355Gly												
ABL1	SNV		COSM12605	p.Phe359Val												
ABL1	SNV		COSM49071	p.Leu384Met												
ABL1	SNV		COSM12604	p.His396Arg												
FOXL2	SNV		COSM33661	p.Cys134Trp												
NOTCH1	SNV		COSM87862	p.Ser2471Ter												
NOTCH1	SNV		COSM13061	p.Gln2440Ter												
NOTCH1	SNV		COSM13048	p.Leu1678Pro												
NOTCH1	SNV		COSM308587	p.(=)												
NOTCH1	SNV		COSM12771	p.Leu1600Pro												
NOTCH1	SNV		COSM13053	p.Arg1598Pro												
NOTCH1	SNV		COSM13042	p.Leu1593Pro												
NOTCH1	SNV		COSM12772	p.Leu1574Pro												

BRAF	SNV		COSM476	p.Val600Glu
BRAF	SNV		COSM471	p.Leu597Arg
BRAF	SNV		COSM467	p.Asp594Gly
BRAF	SNV		COSM462	p.Asn581Ser
BRAF	SNV		COSM6262	p.Arg444Trp
EZH2	SNV		COSM37028	p.Tyr646Phe
CSF1R	SNV		COSM947	p.Tyr969Cys
CSF1R	SNV		COSM310349	p.Ala960Thr
FBXW7	SNV		COSM34016	p.Arg484Met
FBXW7	SNV		COSM22974	p.Arg479Gln
FBXW7	SNV		COSM22965	p.Arg465His
FBXW7	SNV		COSM22986	p.Trp446Ter
FBXW7	SNV		COSM161024	p.Arg441Leu
FBXW7	SNV		COSM22973	p.Arg393Ter
PIK3CA	SNV		COSM1420738	p.(=)
PIK3CA	SNV		COSM1041454	p.(=)
PIK3CA	SNV		COSM27497	p.Arg108His
PIK3CA	SNV		COSM13570	p.Lys111Glu
PIK3CA	SNV		COSM125368	p.Arg115Leu
PIK3CA	SNV		COSM21462	p.Thr324Ile
PIK3CA	SNV		COSM353193	p.(=)
PIK3CA	SNV		COSM754	p.Asn345Lys
PIK3CA	SNV		COSM1420804	p.Ser405Pro
PIK3CA	SNV		COSM757	p.Cys420Arg
PIK3CA	SNV		COSM1420828	p.Asn457Ser
PIK3CA	SNV		COSM759	p.Pro539Arg
PIK3CA	SNV		COSM760	p.Glu542Lys
PIK3CA	SNV		COSM763	p.Glu545Lys
PIK3CA	SNV		COSM1420865	p.Glu547Gly
PIK3CA	SNV		COSM778	p.His701Pro
PIK3CA	SNV		COSM769	p.Cys901Phe
PIK3CA	SNV		COSM770	p.Phe909Leu
PIK3CA	SNV		COSM328026	p.Glu1037Gly
PIK3CA	SNV		COSM775	p.His1047Arg
IDH1	SNV		COSM28746	p.Arg132His

IDH1	SNV		COSM1404902	p.Ile130Val
IDH1	SNV		COSM96922	p.Gly123Arg
ERBB4	SNV		COSM48362	p.Asp931Tyr
ERBB4	SNV		COSM169572	p.Glu928Ter
ERBB4	SNV		COSM232263	p.Arg612Gln
ERBB4	SNV		COSM573362	p.Pro610Thr
ERBB4	SNV		COSM1405173	p.Asp595Gly
ERBB4	SNV		COSM1614287	p.(=)
ERBB4	SNV		COSM110095	p.Ser341Leu
ERBB4	SNV		COSM573356	p.Asp335Tyr
ERBB4	SNV		COSM1405181	p.(=)
ERBB4	SNV		COSM160825	p.His295Gln
ERBB4	SNV		COSM1015994	p.His277Asn
ERBB4	SNV		COSM1251447	p.Tyr268Ter
ERBB4	SNV		COSM1405184	p.Thr244Ala
ERBB4	SNV		COSM573353	p.Ala235Val
ERBB4	SNV		COSM48369	p.Asn181Ser
ERBB4	SNV		COSM442267	p.Pro172Arg
CSF1R	INDEL		AMXsynt5	p.?
RB1	INDEL		COSM870	p.L676fs*16
CDH1	INDEL		AMXsynt10	p.G81_F91del
KDR	INDEL		AMXsynt2	p.V1199fs*27
GNAQ	INDEL		AMXsynt8	p.Met227_Phe228d
EGFR	INDEL		COSM6223	p.Glu746_Ala750de
PTEN	INDEL		COSM4956	p.Tyr76fs
PTEN	INDEL		COSM4978	p.Met198del
PTEN	INDEL		COSM4936	p.Phe347fs
APC	INDEL		COSM41617	p.Ser1234fs
APC	INDEL		COSM18719	p.Glu1309fs
APC	INDEL		COSM18993	p.Arg1399fs
APC	INDEL		COSM13864	p.Ser1465fs
APC	INDEL		COSM18561	p.Thr1556fs
ABL1	INDEL		COSM235737	p.Ile293delinsMetP
NPM1	INDEL		COSM17559	p.Trp288fs
RET	INDEL		COSM1048	p.Glu632_Thr636de

VHL	INDEL		COSM17886	p.P99fs*60												
VHL	INDEL		COSM14412	p.G144fs*15												
PIK3CA	INDEL		COSM12464	p.N1068fs*4												
KIT	INDEL		COSM1326	p.Ser501_Ala502ins												
KDR	INDEL		AMXsynt3	p.?												
FLT3	INDEL		COSM27907	p.Asp600_Leu601in												
TP53	INDEL		COSM85573	p.P27fs*17												
ERBB2	INDEL		COSM20959	p.Glu770_Ala771in												
SMAD4	INDEL		COSM14217	p.T259fs*4												
SMAD4	INDEL		COSM14223	p.Gln410fs												
STK11	INDEL		COSM21212	p.E57fs*7												
SMARCB	INDEL		COSM51386	p.L191fs*26												
SMARCB	INDEL		COSM1057	p.P383fs												
	variants excluded from analysis due to long indel, strand bias or excessive deviation of observed AF from expected AF															
