# Supplementary material for: Validation and Clinical Applications of a Comprehensive Next Generation Sequencing System for Molecular Characterization of Solid Cancer Tissues
Source: Front Mol Biosci. 2019 Sep 25;6:82. doi: 10.3389/fmolb.2019.00082 (PMC6798036; doi:10.3389/fmolb.2019.00082)
Supplement: Supplementary file 7 [file Data_Sheet_7.pdf]

Table S7. Assessment of LOD for detection of Fusions

Run	RNA Samples	Fusion detected	Fusion Read Counts	
			Replicate 1	Replicated 2
**LOD1**	HD231 (50%)	EML4(13)-ALK(20)	50399	58566
**LOD2**	HD231 (20%)	EML4(13)-ALK(20)	50611	59543
**LOD3**	HD231 (10%)	EML4(13)-ALK(20)	49988	41248
**LOD4**	HD231 (5%)	EML4(13)-ALK(20)	20439	21289
**LOD5**	HD231 (2.5%)	EML4(13)-ALK(20)	21197	22666
**LOD6**	HD231 (1%)	EML4(13)-ALK(20)	21995	10329
