# Supplementary material for: Validation and Clinical Applications of a Comprehensive Next Generation Sequencing System for Molecular Characterization of Solid Cancer Tissues
Source: Front Mol Biosci. 2019 Sep 25;6:82. doi: 10.3389/fmolb.2019.00082 (PMC6798036; doi:10.3389/fmolb.2019.00082)
Supplement: Supplementary file 11 [file Data_Sheet_11.pdf]

Table S11A. Reproducibility Summary for SNVs and InDels

Sample	Gene Symbol	COSM ID	Variant	Expected AF (%)	Position	RPD-1	RPD-2	RPD-3
Horizon Dx Quantitative Multiplex	BRAF	COSM476	V600E	10.5	140453136	Yes	Yes	Yes
	KIT	COSM1314	D816V	10	55599321	Yes	Yes	Yes
	EGFR	COSM6224	L858R	3	55259515	No	No	No
	EGFR	COSM28517	E746-A750del	2	55242465	No	No	No
	EGFR	COSM6240	T790M	1	55249071	No	No	No
	EGFR	COSM6252	G719S	24.5	55241707	Yes	Yes	Yes
	KRAS	COSM532	G13D	15	25398281	Yes	Yes	Yes
	KRAS	COSM521	G12D	6	25398284	Yes	Yes	Yes
	NRAS	COSM580	Q61K	12.5	115256530	Yes	Yes	Yes
	PIK3CA	COSM775	H1047R	17.5	178952085	Yes	Yes	Yes
	PIK3CA	COSM763	E545K	9	178936091	Yes	Yes	Yes
	NOTCH1	COSM132827	P668S	31.5	139409754	Yes	Yes	Yes
	APC	-	R2714C	33	112179431	Yes	Yes	Yes
	NF2	COSM1349303	P275fs	8	30060990	No	No	No

Table S11B. Reproducibility summary for CNVs

Sample	Gene Symbol	Position	Location	Expected Copy Number	RPD-4	RPD-5
Horizon Dx Structural Multiplex	MET	116313479	chr7	4.5	3.18*	3.42*
	MYCN	16080662	chr2	9.5	8.98	9.74
	MYC	128748884	chr8	9.8	6.95	6.74
	*: Not a confident somatic CNV call [Minimum Ploidy Gain (5% CI) over expected >= 1.0 OR Minimum Ploidy Loss (95% CI) under expected >= 1.0]					

Table S11C. Reproducibility summary for Fusion trancripts

Run	RNA Samples	Expected Fusion AF	Detected
**RPD-1**	HD640 (2.5%)	CCDC6(1)-RET(12) (2.5%)	Yes
**RPD-2**	HD640 (2.5%)	CCDC6(1)-RET(12) (2.5%)	Yes
**RPD-3**	HD640 (2.5%)	CCDC6(1)-RET(12) (2.5%)	Yes
**RPD-4**	RNA multiplex 5	EML4-ALK (1.5%)	Yes
		CCDC6-RET (1.5%)	Yes
		SLC34A-ROS1 (0.75%)	Yes
**RPD-5**	RNA multiplex 5	EML4-ALK (1.5%)	Yes
		CCDC6-RET (1.5%)	Yes
		SLC34A-ROS1 (0.75%)	Yes
