# Supplementary material for: Validation and Clinical Applications of a Comprehensive Next Generation Sequencing System for Molecular Characterization of Solid Cancer Tissues
Source: Front Mol Biosci. 2019 Sep 25;6:82. doi: 10.3389/fmolb.2019.00082 (PMC6798036; doi:10.3389/fmolb.2019.00082)
Supplement: Supplementary file 13 [file Data_Sheet_13.pdf]

Table S13. Ethanol interference impact on library concentration

DNA Sample	[Library] (pM)	RNA Sample	[Library] (pM)
**RM8398 20% EtOH**	5.0	HD615 (63%) 20% EtOH	8.8
**RM8398 10% EtOH**	4.0	HD615 (63%) 10% EtOH	2,029.6
**RM8398 5% EtOH**	126.6	HD615 (63%) 5% EtOH	3,825.0
**RM8398 1% EtOH**	1,055.8	HD615 (63%) 1% EtOH	3,508.2
**RM8398- Clean**	1,042.0	HD615 (63%)	3,448.2
**HC-C511 20% EtOH**	3.6	RNA multiplex 20% EtOH	2.0
**HC-C511 10% EtOH**	55.6	RNA multiplex 10% EtOH	1.6
**HC-C511 5% EtOH**	715.2	RNA multiplex 5% EtOH	36.8
**HC-C511 1% EtOH**	2,923.2	RNA multiplex 1% EtOH	1,611.2
**HC-C511**	3,307.6	RNA multiplex	762.4
