# Supplementary material for: Validation and Clinical Applications of a Comprehensive Next Generation Sequencing System for Molecular Characterization of Solid Cancer Tissues
Source: Front Mol Biosci. 2019 Sep 25;6:82. doi: 10.3389/fmolb.2019.00082 (PMC6798036; doi:10.3389/fmolb.2019.00082)
Supplement: Supplementary file 14 [file Image_1.pdf]

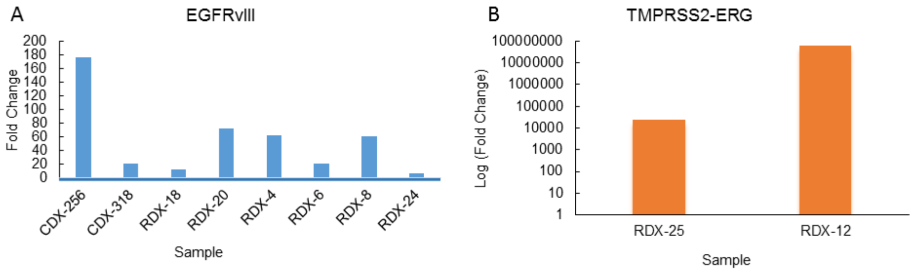

Figure S1. Confirmation of *EGFRvIII* (A) and *TMPRSS2-ERG* (B) fusion detected by the OCAv1 in solid tumor samples using TaqMan qRT-PCR fusion assays. Relative expression of fusion transcripts was quantified by the $\Delta\Delta^{Ct}$ method using *GAPDH* as a reference and a negative sample without fusions as the calibrator and is shown as fold changes (A) or $\log_{10}$(fold change) (B). Replicates of experiments were performed when sufficient RNA was available.
